# Non-van-der-Waals Oriented Two-Dimensional UiO-66 Films by Rapid Aqueous Synthesis at Room Temperature

**DOI:** 10.1021/jacs.4c11134

**Published:** 2025-02-20

**Authors:** Heng-Yu Chi, Shuqing Song, Kangning Zhao, Kuang-Jung Hsu, Qi Liu, Yueqing Shen, Anne Faustine Sido Belin, Arthur Allaire, Ranadip Goswami, Wendy L. Queen, Kumar Varoon Agrawal

**Affiliations:** † Laboratory of Advanced Separations, École Polytechnique Fédérale de Lausanne (EPFL), 1950 Sion, Switzerland; ‡ Laboratory for Functional Inorganic Materials, École Polytechnique Fédérale de Lausanne (EPFL), 1950 Sion, Switzerland

## Abstract

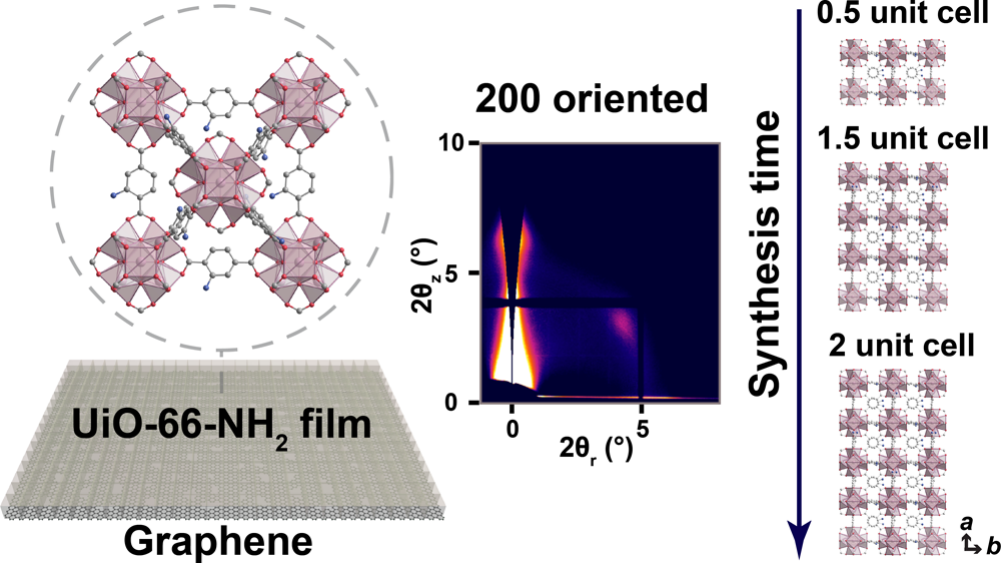

The synthesis of MOFs in a two-dimensional (2D) film morphology is attractive for several applications including molecular and ionic separation. However, 2D MOFs have only been reported from structures that crystallize in lamellar morphology, where layers are held together by van der Waals (vdW) interaction. By comparison, UiO-66, one of the most studied MOFs because of its exceptional chemical stability, has only been reported in three-dimensional (3D) morphology. 2D UiO-66 is challenging to obtain given the robust isotropic bonds in its cubic crystal structure. Herein, we report the first synthesis of non-vdW 2D UiO-66-NH_2_ by developing crystal growth conditions that promote in-plane growth over out-of-plane growth. Continuous, oriented UiO-66-NH_2_ film with thickness tunable in the range of 0.5 to 2 unit cells could be obtained by sustainable, scalable chemistry, which yielded attractive ion–ion selectivity. The preparation of non-vdW 2D MOF is highly attractive to advance the field of MOF films for diverse applications.

## Introduction

Metal–organic frameworks (MOFs) are a class of porous materials known for high surface area as well as highly tunable porous structures, topologies, and functionalities.^1,2^ They are synthesized by coordination of a metal or metal oxide node with an organic linker. The relative ease of synthesis and the degree of tunability have made MOFs a material of choice for diverse applications including gas storage^3,4^ and separations,^5,6^ sensing,^7,8^ catalysis,^9,10^ and biochemical applications.^11,12^ While most MOFs have three-dimensional (3D) morphology, several MOFs have been reported in two-dimensional (2D)^13−15^ and thin platelet^16,17^ morphologies. 2D MOFs are synthesized in lamellar sheets, where it has been shown that these layers can be exfoliated into monolayers, which are attractive as a building block of thin films.^13,18,19^

UiO-66, named after the University of Oslo,^20^ is one of the most studied MOFs thanks to its exceptional chemical and thermal stability attributed to a strong Zr–O bond and a high coordination number of Zr.^21,22^ UiO-66 is formed by the coordination of the zirconium-oxo cluster with terephthalic acid where an octahedral cage shares its eight triangular faces with eight tetrahedral cages (Figure S1), each featuring an Å-scale triangular aperture. The pore size of UiO-66 is attractive to differentiate molecules and ions. Thanks to its high stability, including in water, UiO-66 films have been explored for gas separation,^23,24^ desalination,^25,26^ and ion–ion separation.^27−29^

UiO-66 possesses a 3D structure. Its synthesis in 2D morphology has not been reported, mainly because of the challenges from the isotropic robust bonds in its structure. While an emerging class of non-van der Waals (non-vdW) quasi-2D materials has been synthesized from nonlayered 3D crystals,^30−33^ they have been predominantly demonstrated for transition metal oxides. Extending this concept to MOFs, and in particular UiO-66, is highly attractive to prepare 2D morphologies and ultrathin films for advancing thin film applications for highly stable MOFs. A potential approach is anisotropic 2D growth of UiO-66; however, this has not been reported. As a result, the state-of-the-art UiO-66 films are 2–3 orders of magnitude thicker than the desired 2D film (Figure S2 and Table S1). Some oriented surface-coordinated MOFs (SURMOFs) thin films have been successfully achieved;^34−37^ however, the thickness of these SURMOF films generally exceeds 100 nm, and fabricating a nm-thick dense structure for membrane applications remains a significant challenge.

UiO-66 films in the literature suffer from limitations such as misoriented grains with limited control over grain morphology, size, and orientation.^25,26^ Their synthesis often involves organic solvent, high temperature, and a long synthesis time. Synthesis of an oriented 2D film with a large grain size (≥1 μm) will reduce grain-boundary defects and improve the film’s overall quality for application in molecular and ionic separation. Such a method will allow for control over film thickness, with the ability to tune the thickness down to a few nanometers.

Herein, we report the first non-vdW 2D MOF in the form of a 2D UiO-66-NH_2_ film by developing conditions leading to anisotropic 2D growth. Briefly, the composition of the precursor solution was screened to develop growth conditions that delay homogeneous nucleation of UiO-66-NH_2_ in the bulk solution, promote the in-plane growth of 2D nuclei, and slow out-of-plane growth. This was further facilitated by the use of atomically smooth graphitic substrates. A crystallographic registry between the graphene superstructure and the 2D lattice of UiO-66 along the *a*-out-of-plane direction was observed. The thickness of the film could be tuned from 0.5 to 2 unit cells. Pinhole-free sub-10 nm-thick films could be prepared, evident from K^+^/Mg^2+^ selectivity. Thanks to the high stability of the UiO-66 framework, unit-cell-thick films were stable in 0.1 M salt solutions for more than one month. The chemistry for the synthesis of the film is green (water as opposed to organic solvent) and user-friendly, involving a room-temperature synthesis reaction time that is only a few minutes.

## Results and Discussion

**Figure 1:**
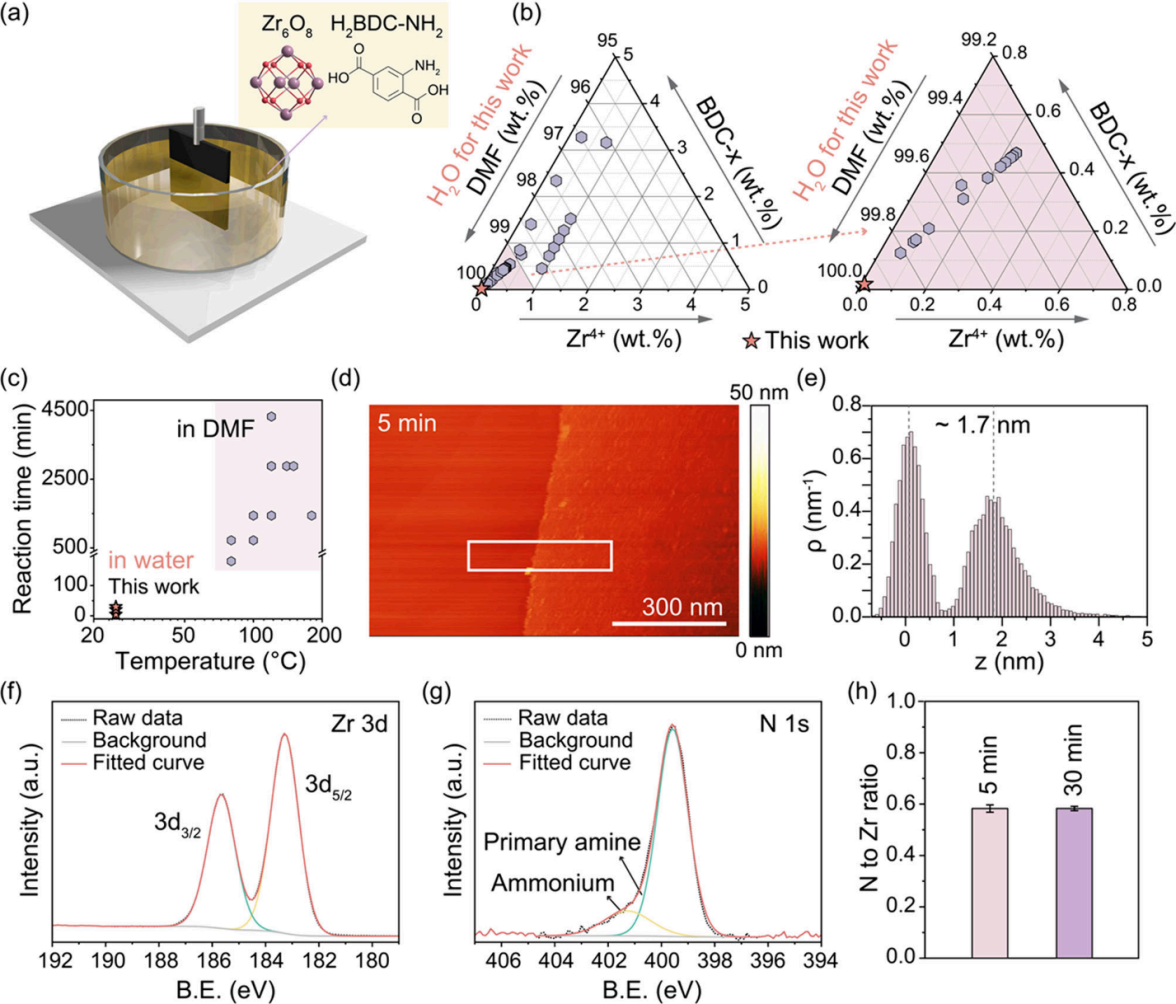
(a) Schematic of non-vdW 2D UiO-66-NH_2_ film synthesis. (b) Composition diagram comparing the Zr^4+^ and BDC-*x* concentration used in this study and those reported in the literature (Table S4), where *x* can be NH_2_ or H. (c) Comparison of reaction time and temperature used in this study and those reported in the literature (Table S3). (d) AFM image and (e) the corresponding height profile of 5 min growth of the non-vdW 2D UiO-66-NH_2_ film, acquired from the white box in (d). (f) Zr 3d and (g) N 1s XPS spectra from non-vdW 2D UiO-66-NH_2_ films with a growth time of 5 min. (h) The ratio of N to Zr of non-vdW 2D UiO-66-NH_2_ films. The error bars were calculated from the standard deviation of XPS measurements from three different regions.

UiO-66-NH_2_, formed by the coordination of a hexanuclear zirconium-oxo cluster and 2-aminoterephthalate (BDC-NH_2_), possesses a cubic structure with a composition of Zr_6_O_6_(BDC-NH_2_)_6_.^20,38^ It has been reported that crystallinity can be enhanced with modulators such as hydrochloric acid,^39^ benzoic acid,^40^ formic acid,^41^ and acetic acid.^42^ Acetic acid, with its relatively high p*K*_a_, is a weaker acid compared to other commonly used modulators. This results in a slower deprotonation and nucleation rate, which is essential for promoting in-plane growth of the 2D films while maintaining an enhanced crystallinity. Furthermore, the slower deprotonation contributes to a lower defect density, improving film quality. To address this, synthesis conditions were modulated by screening different amounts of acetic acid as a modulator to improve crystallinity, various metal and ligand concentrations, metal-to-ligand ratio, and pH. Importantly, water was used as a solvent, keeping sustainability in mind. Atomically smooth graphitic substrates were used to promote the in-plane growth. To form a thin film, substrates were immersed vertically halfway into the synthesis solution (Figure 1a), ensuring a distinct interface between the substrate and thin film. The film near the interface was then characterized by scanning electron microscopy (SEM) and atomic force microscopy (AFM). These experiments allowed us to screen conditions for the successful growth of a thin film. Precursor concentration exceeding 1 mM and pH higher than 3.5 led to the deposition of aggregated particles instead of a uniform film (Figure S3). An increase in the ligand-to-metal ratio in the range of 8–10 accelerated undesired homogeneous nucleation in the bulk solution, resulting in rapid precipitation within 10 min and deposition of particles (Figure S4).

The optimal conditions found using the above screening route was ultradilute precursor solution consisting of Zr^4+^ and BDC-NH_2_ at an equimolar concentration of 1 mM, acetic acid concentration of 108 mM, and a solution pH of 3.3 (Table S2). This delayed homogeneous nucleation and no crystals were formed in the bulk for up to 30 min. This strategy of delaying the reaction kinetics within a relatively low pH regime deviates from the usual approach to maximize the yield of crystalline UiO-66-NH_2_ involving a synthesis solution pH of ∼4.3.^43^ Within 5 min of synthesis at room temperature in aqueous solution, high-quality, continuous, nanometer-thick UiO-66-NH_2_ films (see discussion below) could be obtained on highly ordered pyrolytic graphite (HOPG) or graphene film as a substrate, as indicated by AFM analysis (Figure 1d,e).

Compared to film fabrication techniques reported in the literature, this approach is highly attractive. First, the method yields nanometer-thick film compared to the state-of-the-art, where it is challenging to reduce the thickness below 1000 nm (Figure S2 and Table S1). Second, this method of preparing a film is sustainable. The use of water as a solvent is environmentally friendly compared to organic solvent (dimethylformamide, DMF), which is the solvent of choice in the literatures to form UiO-66 film. Synthesis is carried out at room temperature in a few minutes in contrast to the literature, where the synthesis of UiO-66-x films typically occurs at temperatures above 80 °C and requires more than 3 h to form a continuous film (Figure 1c and Table S3). Finally, the approach utilizes one of the lowest precursor concentrations for synthesizing UiO-66-*x* thin films (Figure 1b and Table S4), where *x* can be NH_2_ or H.

The chemical compositions of the deposited films were analyzed through X-ray photoelectron spectroscopy (XPS). The binding energies (BEs) for Zr 3d_5/2_ and Zr 3d_3/2_ were centered at 183.2 and 185.6 eV, respectively, with a peak splitting of 2.4 eV (Figures 1f, S5a). Two N 1s peaks at 399.6 and 401.3 eV were observed (Figures 1g, S5b), corresponding to the primary amine in BDC-NH_2_ and the ammonium ion formed from the reaction between the primary amine and H_2_O,^44,45^ respectively. These BEs are consistent with the literature on UiO-66-NH_2_,^46,47^ indicating that the chemical environments of Zr and N within the nanometer-thick films were similar to that in the bulk crystal (Supplementary Note 1, Figure S6). The XPS data, collected from three separate regions on the thin film, were consistent, indicating that the film was homogeneous in composition (Figure S7). As expected, Zr and N were not present in the HOPG substrate (Figure S8).

The N/Zr atomic ratio in the UiO-66-NH_2_ framework serves as an indicator of the linker vacancy defect density because N originates from the ligand. A defect-free crystal should have a ratio of 1. XPS (Figure 1h and Supplementary Note 2) indicated a ratio of approximately 0.58 in the non-vdW 2D UiO-66-NH_2_ films prepared with growth times of 5 to 30 min, highlighting the presence of significant vacancy defects within the films. To quantify the defect ratio in non-vdW 2D UiO-66-NH_2_ films, TGA analysis was performed. As shown in Figures S9 and S10 and detailed in Supplementary Note 3, the TGA results indicate a linker vacancy defect density of approximately 30% (Table S5). The linker defect is attributed to the use of acetic acid as a modulator, which competes with the linker to coordinate with the zirconium-oxo cluster.^41,42,48^ This defect density in this study agrees well with literature data of 30–45% for UiO-66 synthesized in the presence of a modulator.^21,49^ The presence of a linker defect is not expected to alter the crystal structure because of an overall high coordination number.^21,22^

**Figure 2:**
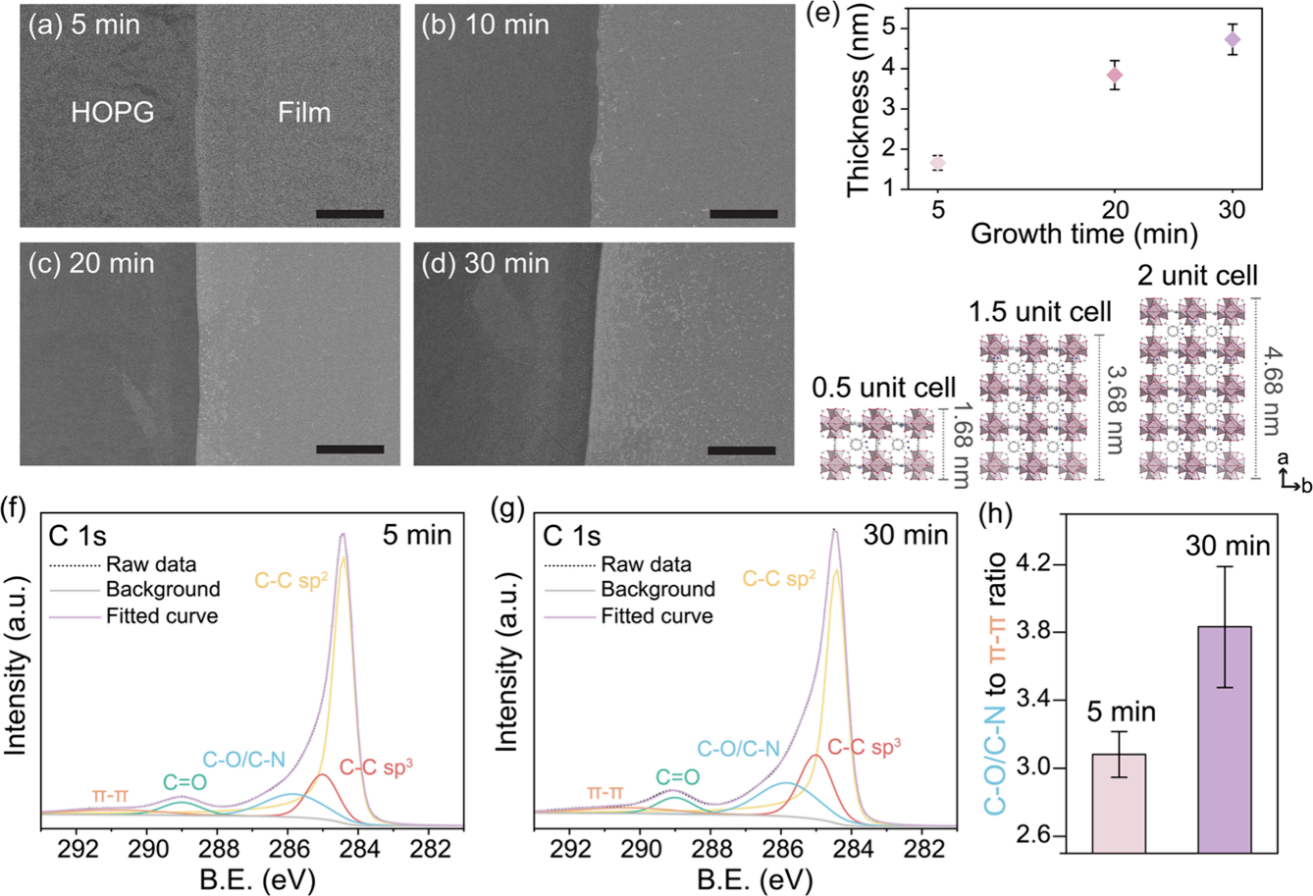
(a–d) SEM images of non-vdW 2D UiO-66-NH_2_ films (scale bar: 25 μm): (a) 5 min, (b) 10 min, (c) 20 min, (d) 30 min. (e) Half to two unit-cell thick non-vdW 2D UiO-66-NH_2_ films on HOPG versus growth time. The error bars were calculated from the standard deviation of 3–5 thickness measurements. (f, g) C 1s XPS spectra from non-vdW 2D UiO-66-NH_2_ films with a growth time of (f) 5 min and (g) 30 min. (h) The ratio of C–O/C–N to π–π* transition signals of non-vdW 2D UiO-66-NH_2_ films. The error bars were calculated from the standard deviation of the XPS measurements from three different regions.

The thickness of the UiO-66-NH_2_ films could be controlled by varying the synthesis time from 5 to 30 min. Uniform contrast in SEM images indicated a continuous and uniform film (Figure 2a–d). Notably, the film covers the entire substrate, as shown by SEM (Figure S11) and optical microscopy (Figure S12) images from both localized and large-area analyses. High-magnification SEM images further confirm that the film is free of pinholes (Figure S13). AFM analysis revealed that films synthesized in 5, 20, and 30 min had thicknesses of 1.7 ± 0.2 nm (Figure 1d and e), 3.8 ± 0.4 nm (Figure S14a and b), and 4.7 ± 0.4 nm, respectively (Figure S14c and d). The height profile of the non-vdW 2D UiO-66-NH_2_ film (Figure S15) reveals a continuous film with a consistent thickness across the surface and a distinct gap from the substrate, indicating uniform film height without pinhole defects. To verify uniformity, we examined the height profiles at intentionally created cracks on graphene (Figure S16) formed during the transfer process. The 5 min-grown non-vdW 2D UiO-66-NH_2_ film exhibited a smooth surface with a consistent thickness of 1.7–1.8 nm across areas away from the interface (Figure S17), in agreement with the thickness observed on the HOPG substrate. Surface roughness analysis revealed a relatively low RMS value (<1.6 nm) for the non-vdW 2D UiO-66-NH_2_ film with growth times between 5 and 30 min (Figures S14 and S15). The mechanical strength of the non-vdW 2D UiO-66-NH_2_ film was assessed using the modulus map in AFM measurements. The 30 min-grown film exhibits a Young’s modulus of 4.3 ± 0.3 GPa, which makes it mechanically robust (Figure S18).

The thicknesses of the non-vdW 2D UiO-66-NH_2_ films grown for 5, 20, and 30 min correspond to 0.5, 1.5, and 2 unit cells of UiO-66-NH_2_ along the *a*-out-of-plane direction (Figures 2e and S19, Supplementary note 4). A 0.5 unit cell results from bisecting the face-centered-cubic unit cell of UiO-66-NH_2_, as illustrated in Figure 2e. We hypothesize that the film terminates at the metal node mainly because this would enable a strong cation−π interaction^50^ between the cationic hexanuclear Zr cluster and the π system in the crystalline graphene lattice. The hypothesis is supported by the high density of linker vacancy defects. For example, if one removes linkers on the top and bottom faces of a 0.5-unit-cell-thick film, it would result in ∼33% defect density, consistent with the high defect density in experimental data.

XPS C 1s spectra identified a BE of 285.8 eV associated with C–O or C–N bonds originating from UiO-66-NH_2_ (Figures 2f and g). A BE of 290.3 eV was also observed, which can be attributed to the π–π* transition signal in the HOPG substrate. This was confirmed by the analysis of a bare HOPG substrate (Figure S20). Therefore, the π–π* transition signal from the substrate was used as a reference for comparative analysis. This allowed the quantification of C–O/C–N content (285.8 eV) for films grown for 5 and 30 min, yielding an average thicknesses of 1.7 and 4.7 nm, respectively. As expected, the ratio of C–O/C–N to π–π* transition signals increased for the thicker film (Figure 2h), aligning with the accumulation of a larger quantity of UiO-66-NH_2_ in the thicker film with an XPS detection depth resolution of 5–10 nm from the surface.^51^

The porosity of non-vdW 2D UiO-66-NH_2_ was evaluated through N_2_ adsorption at 77 K. A significant increase in N_2_ adsorption was observed for non-vdW 2D UiO-66-NH_2_ on graphite compared to standalone graphite (Figure S21 and Supplementary note 3). The estimated surface area was 707.38 m^2^/g, with a corresponding pore volume of 0.676 cm^3^/g based on the quantity of non-vdW 2D UiO-66-NH_2_ on graphite.

**Figure 3:**
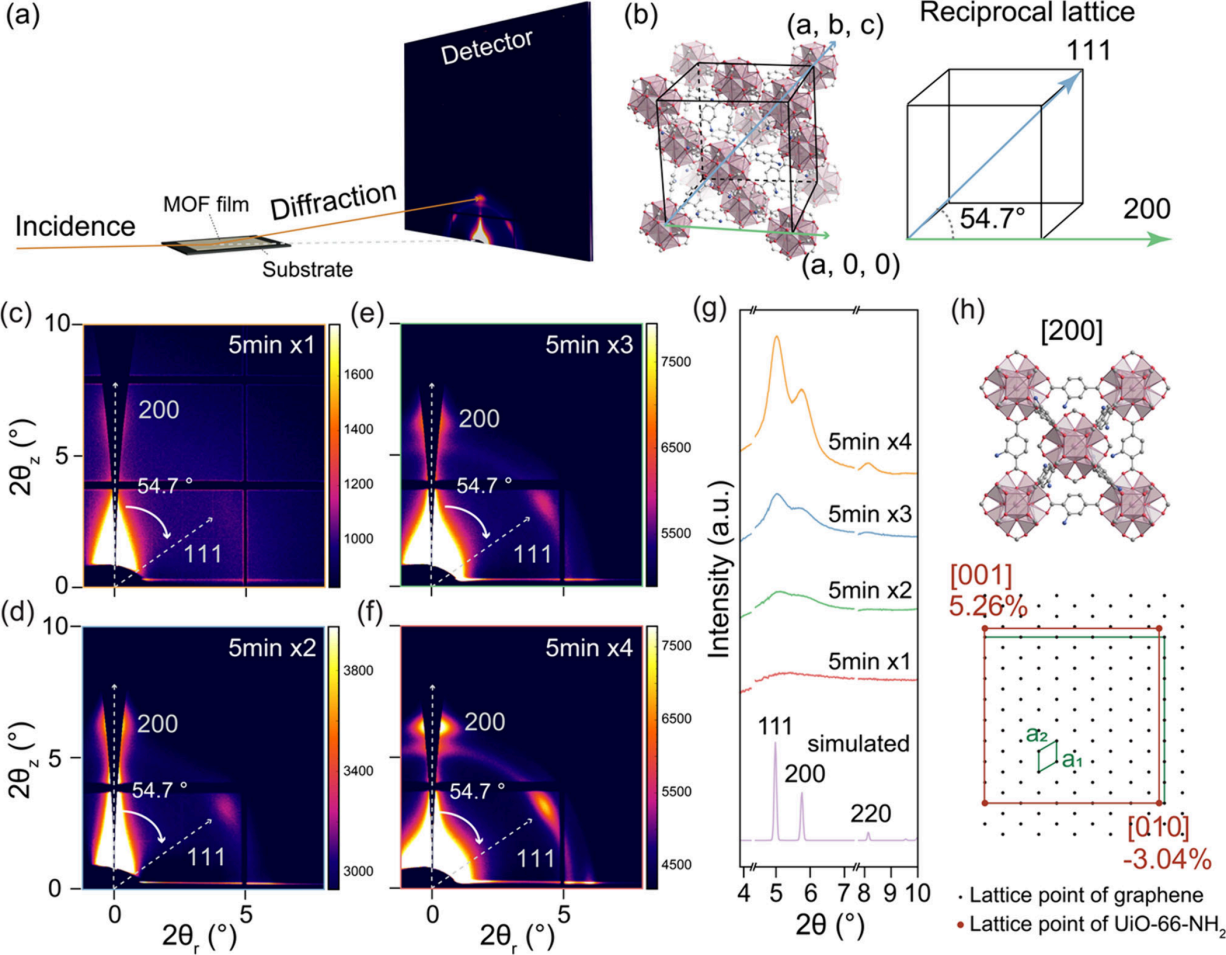
(a) Schematic of the measurement geometry of GIWAXS. (b) The structural model of cubic UiO-66-NH_2_ in real space and reciprocal space indicates the relation between 111 and 200 facets. (c–f) GIWAXS images from non-vdW 2D UiO-66-NH_2_ films prepared in different growth cycles: (c) 5 min × 1 (cycle 1), (d) 5 min × 2 (cycle 2), (e) 5 min × 3 (cycle 3), and (f) 5 min × 4 (cycle 4). (g) The integrated 1D patterns from GIWAXS images. (h) Crystallography registry between unit cell of UiO-66-NH_2_ and a supercell of graphene.

The presence of order in the as-deposited films was probed by synchrotron-based grazing incidence wide-angle X-ray scattering (GIWAXS). For this, UiO-66-NH_2_ films were deposited on chemical vapor deposition (CVD)-derived graphene resting on SiO_2_-coated Si wafers (SiO_2_/Si). The analysis was carried out for films prepared with 5 min of deposition time. Three other thicker films were also analyzed by repeating the deposition to 4 cycles. These are termed cycle 2, cycle 3, and cycle 4. These samples were subjected to GIWAXS measurements at a small incidence angle range of 0.08–0.10°, identified as the critical angle to yield the highest intensity, ensuring that reflections fully came from the thin films (Figures 3a and S22, Supplementary note 5). The grazing incidence footprint at these angles was approximately 2.1 to 2.6 cm across the samples, with a beam width of 124 μm (Figure S23 and Table S6), allowing for the acquisition of structural data across a large film area (∼3 mm^2^ in size). Additionally, corrections were applied to adjust for the distortions caused by the curvature of the Ewald sphere when projecting onto a flat 2D detector, resulting in the missing wedge in the final GIWAXS images (Supplementary note 6).^52^ The correction enables the accurate extraction of the quantitative structural information.

GIWAXS of the four UiO-66-NH_2_ films revealed broad peaks (Figures 3c–f) attributed to the ultrathin nature. Peak intensity increased monotonically with the thickness of the film from improved scattering in the thicker films. An out-of-plane 200 nm texture was observed, indicating that the films were preferentially oriented along the *a*-out-of-plane direction. This was further corroborated by the scattering intensity along the azimuthal angle, which identified 111 facets oriented ∼54.7° relative to the 200 facets, consistent with the angle between these two lattice planes in the cubic system (Figure 3b, Supplementary note 7). Combined with XPS and AFM analysis, this confirms anisotropic in-plane growth of non-vdW 2D UiO-66-NH_2_ films with a thickness of just a few unit cells.

When films were directly deposited on SiO_2_/Si without a graphene layer (Figure S24a) under identical growth conditions, relatively weak scattering without a preferred orientation was observed (Figure S24b). We also used single-crystal sapphire as a substrate for non-vdW 2D UiO-66-NH_2_ growth. However, we observed no detectable signal from UiO-66-NH_2_ on sapphire even when using the same protocol applied for film growth on graphene (Figure S25). This result highlights the essential and unique role of graphene in facilitating the growth of non-vdW 2D UiO-66-NH_2_ films. Moreover, substrates featuring only a graphene layer on SiO_2_/Si displayed no detectable signals (Figure S26), confirming that the observed signals in the thin films originate from UiO-66-NH_2_. Further analysis of the 1D scattering profiles, achieved by integrating the full scattering signal through GIWAXS (Figures 3g and S27), agreed well with the simulated powder pattern of UiO-66-NH_2_. To elucidate the influence of graphene on the preferred orientation of UiO-66-NH_2_, selected area electron diffraction (SAED, Figure S28) was carried out to investigate the potential lattice registry between UiO-66-NH_2_ and graphene. Analysis revealed that the UiO-66-NH_2_ film oriented along the *a*-out-of-plane direction has only a small lattice mismatch with graphene, −3.04% along the *b*-axis and 5.26% along the *c*-axis (Figure 3h, Supplementary note 8). This analysis utilized the smallest structural unit of UiO-66-NH_2_ to fit on the graphene lattice, suggesting graphene offers the structural registry in facilitating the preferred growth orientation along the 200 lattice plane of UiO-66-NH_2_.

**Figure 4:**
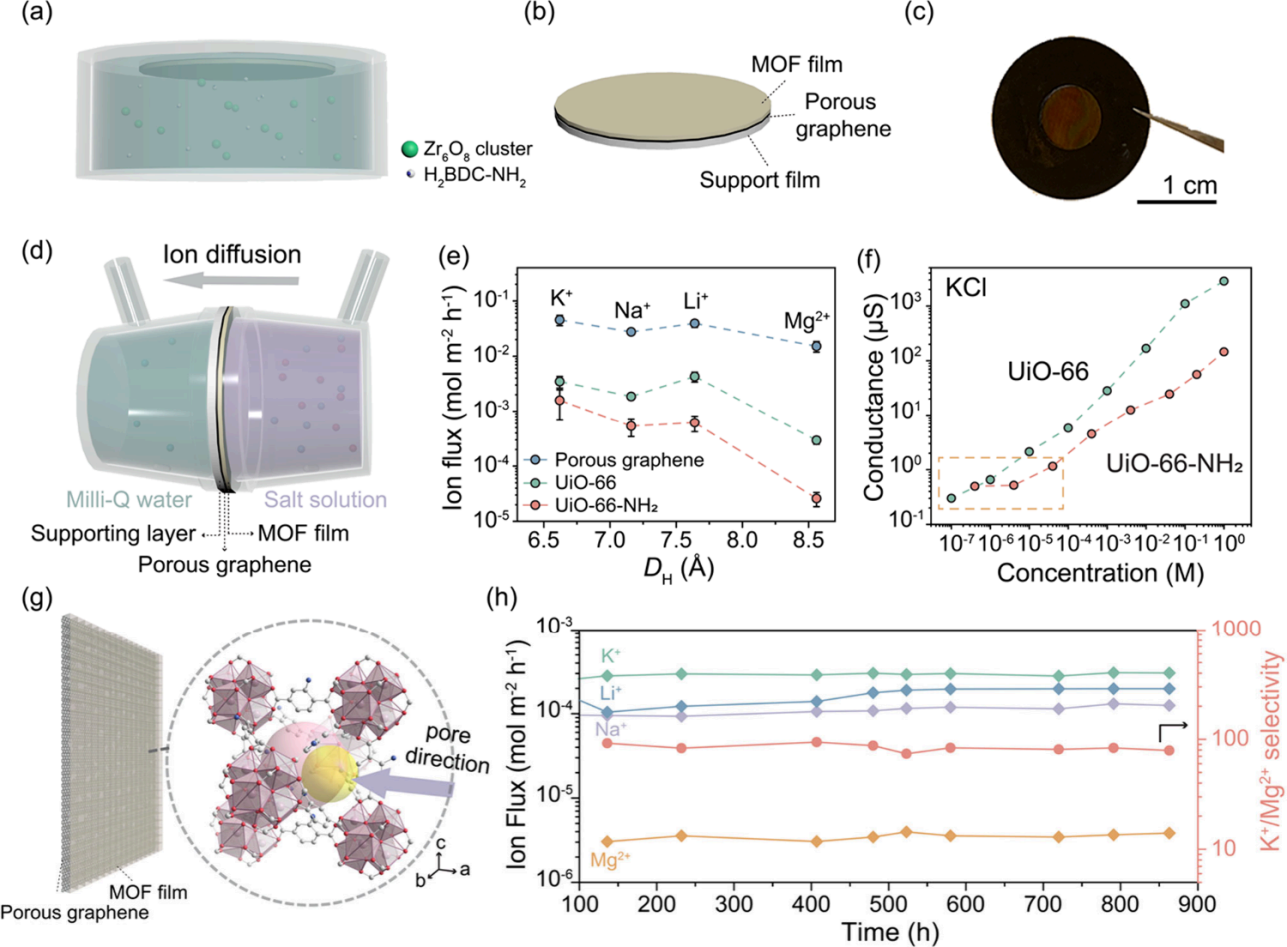
(a) Schematic of the fabrication of a non-vdW 2D UiO-66-NH_2_ film with porous graphene facing downward, directly in contact with the synthesis solution. (b) Schematic of a UiO-66-NH_2_ film on porous graphene reinforced with a support film. (c) Photograph of the film on an annular gasket. (d) Schematic of an H-type cell demonstrating an ion diffusion experiment across a non-vdW 2D UiO-66-NH_2_ film. (e) Ion flux of K^+^, Na^+^, Li^+^, Mg^2+^ versus their *D*_H_ across porous graphene, UiO-66, and UiO-66-NH_2_. The error bars were calculated from the standard deviation for three different films listed in Table S7. (f) Concentration-dependent conductance of K^+^ of non-vdW 2D UiO-66-NH_2_ and UiO-66 films. The box indicated the region with saturated conductance of UiO-66-NH_2_ at low concentration. (g) Schematic of the pore direction of the MOF film relative to the preferred oriented 200 facet. (h) Long-term ion diffusion data from non-vdW 2D UiO-66-NH_2_ using an equimolar ion mixture (0.1 M of KCl, NaCl, LiCl, and MgCl_2_).

UiO-66-NH_2_ hosts a triangular pore aperture with a size of ∼6 Å (Figure S1), a dimension well-suited for the selective transport of common salt ions dissolved in water. Moreover, the presence of linker vacancy defects is expected to slightly increase pore aperture, enabling a selective passage of monovalent ions compared to bivalent ions.^53^ To investigate this, 4.7 nm thick non-vdW 2D UiO-66-NH_2_ films were deposited on porous graphene where the latter hosted an average pore size of 2.0 nm (Figure S29), large enough to permeate hydrated ions without selectivity.^54^ The UiO-66-NH_2_ film was deposited by floating porous graphene on top of the synthesis solution (Figure 4a). To ensure that graphene does not crack during the deposition, it was mechanically reinforced with a thin film of nanoporous carbon (NPC)/Nafion (support film) similar to a recent report (Figures 4b and S30).^54^ Films were placed in between two annular gaskets with a diameter of 8 mm (Figure 4c) and were sealed between the two reservoirs of an H-type cell (Figure 4d). The UiO-66-NH_2_ side of the film contacted the 1 M salt solution (feed side), while the other side contacted Milli-Q water (permeate side, Figure 4d, Supplementary note 9).

The flux of K^+^, Na^+^, and Li^+^ ions from the UiO-66-NH_2_ film were up to ∼2 orders of magnitude larger than Mg^2+^ (Figure 4e), resulting in average selectivities of K^+^/Mg^2+^, Li^+^/Mg^2+^, and Na^+^/Mg^2+^ of 61.0, 23.9, and 20.7, respectively, with the highest selectivities for K^+^/Mg^2+^, Li^+^/Mg^2+^, and Na^+^/Mg^2+^ being 99.8, 45.1, and 37.2, respectively (Table S7). This indicates that pinhole-free non-vdW 2D UiO-66-NH_2_ films were successfully synthesized. Control experiments with porous graphene yielded much lower selectivities, with K^+^/Mg^2+^, Li^+^/Mg^2+^, and Na^+^/Mg^2+^ average selectivities of 3.0, 2.6, and 1.8, respectively. This confirms that graphene did not contribute to the selectivity observed from the UiO-66-NH_2_ film, which is consistent with the large pore size in graphene. The average K^+^ flux (1.6 × 10^–3^ mol m^–2^ h^–1^) from UiO-66-NH_2_ was an order of magnitude smaller than that of the porous graphene, indicating that the transport was controlled by the MOF layer (Figure 4e). In addition, the stability of the non-vdW 2D UiO-66-NH_2_ film was tested with a mixed equimolar feed (0.1 M KCl, NaCl, LiCl, and MgCl_2_, Figure 4h), demonstrating a long stability of 863 h (∼36 days, over 5 weeks) in salt environments with stable ion flux and a K^+^/Mg^2+^ selectivity of ∼80.

Notably, in our non-vdW 2D UiO-66-NH_2_ film, linker defects slightly expand the pore aperture^53^ which is located ∼54.7° away from the preferred oriented 200 facet (Figure 4g), permitting monovalent ions to pass while effectively blocking larger divalent ions through size exclusion. Additionally, linker loss within the UiO-66-NH_2_ structure creates open metal sites, which add positive charges to the framework. This increase in positive charge enhances electrostatic repulsion, especially against divalent ions like Mg^2+^, thereby favoring the passage of monovalent ions such as K^+^ due to differential repulsion. The rejection of the divalent Mg^2+^ ion can be further attributed to electrostatic repulsion with the positively charged NH_2_ groups.^55−57^ Non-vdW 2D UiO-66 thin films were also prepared following a similar method (Figures S31–33, Supplementary note 10) to serve as a charge comparison since UiO-66-NH_2_ possesses charged amino functional groups, whereas UiO-66 does not. In this case, average K^+^/Mg^2+^, Li^+^/Mg^2+^, and Na^+^/Mg^2+^ selectivities were restricted to 11.8, 14.5, and 6.3, respectively. The much higher selectivity observed from the 2D UiO-66-NH_2_ film can be attributed to a positively charged functional group in UiO-66-NH_2_.

This effect was further corroborated by the concentration-dependent conductance of K^+^ in the concentration range of 10^–7^ to 1 M. The ion flux exhibited a plateau at low salt concentrations (<10^–5^ M), whereas UiO-66 showed a near-linear relationship with salt concentration (Figure 4f), indicating the contribution of charge from the amino group of UiO-66-NH_2_ compared to UiO-66 (Supplementary note 11).^58,59^

Few MOF membranes have been reported for ion–ion separation using a diffusion-based separation method, and among these, our membrane demonstrates a competitive ion–ion separation performance when compared with Al-MOF and other membranes (Figure S34 and Table S8). It is worth mentioning that, in contrast to ion drift under an external electric field, the diffusion method in the absence of an external field consistently yields a lower ion flux. Further, the pore density in the graphene is small, which limits ion flux. In the future, membranes without a graphene support layer will be investigated.

## Conclusion

Overall, we show the first example of the synthesis of a non-vdW 2D MOF by developing UiO-66 films with tunable thicknesses in the range of 0.5 to 2 unit cells. Remarkably, this was achieved via green, sustainable chemistry, which is also user-friendly, eliminating the need for organic solvents, high-temperature reactors, and long synthesis time. With this approach, oriented UiO-66-NH_2_ films with a thickness of just a few nanometers could be prepared, compared to the mostly μm-thick films in the literature. Pinhole-free non-vdW 2D UiO-66 films were stable in salt solution and yielded attractive ion–ion selectivity. Our work underscores that in-plane growth of non-vdW MOFs can be achieved by careful screening of growth conditions. This highlights the potential to transform non-vdW MOFs to a 2D morphology, which will greatly advance the application of MOF films.

## Supporting Information Available

Additional experimental details, materials, and methods; structural characterization data for non-vdW 2D UiO-66-NH_2_ and UiO-66 films; supporting tables provide comparisons of film thickness, synthesis conditions, and ion flux (PDF)
