# Supplementary material for: Non-van-der-Waals Oriented Two-Dimensional UiO-66 Films by Rapid Aqueous Synthesis at Room Temperature
Source: J Am Chem Soc. 2025 Feb 20;147(9):7255–63. doi: 10.1021/jacs.4c11134 (PMC11887446; doi:10.1021/jacs.4c11134)
Supplement: Supplementary file 1 — ja4c11134_si_001.pdf [file ja4c11134_si_001.pdf]

Non-van-der-Waals oriented two-dimensional UiO-66 films by rapid aqueous synthesis at room temperature

Heng-Yu Chi,[‡] Shuqing Song,[‡] Kangning Zhao, Kuang-Jung Hsu, Qi Liu, Yueqing Shen, Anne Faustine Sido Belin, Arthur Allaire, Ranadip Goswami, Wendy L. Queen and Kumar Varoon Agrawal[*]

Experimental section

◆ Chemicals

Zirconyl chloride octahydrate ($ZrOCl_2·8H_2O$, 98+%), 2-aminoterephthalic acid ($BDC-NH_2$, 99+%), terephthalic acid (BDC, 98+%), sodium hydroxide (NaOH, 98%), and magnesium chloride ($MgCl_2$, 99%) were purchased from Thermal Scientific. Acetic acid (CH_3COOH, 99-100%), iron(III) chloride ($FeCl_3$, 97%), sodium persulfate ($Na_2S_2O_8$, 98%), lithium chloride (LiCl, 99%), sodium chloride (NaCl, 99%), D-(+)-turanose (98%), dimethylformamide (DMF, 99.8%), heptane (99%), and Nafion™ solution (5wt.% in lower aliphatic alcohols and water) were purchased from Sigma-Aldrich. Potassium chloride (KCl, 99%) was purchased from Alfa Aesar. Poly(1-trimethylsilyl-1-propyne) (PTMSP) and poly(styrene-b-4-vinyl pyridine) (PS-b-P4VP, M_w/M_n=1.05) were obtained from ABCR and Polymer Source, respectively. Hydrochloride acid (HCl, 32% solution in water), toluene, and acetone were purchased from Acros Organics, Merk, and Thommen-Furle AG, respectively. Graphite nanoplatelets were purchased from Thermo Scientific (thickness of 2-10 nm). All chemicals were used without further purification.

◆ Synthesis of non-vdW UiO-66-NH$_2$ or UiO-66 films

The preparation of synthesis precursors for UiO-66-NH$_2$ and UiO-66 thin film fabrication was adapted with modifications from the crystalline powder synthesis method proposed by Huelsenbeck *et al.*[1] First, 2.58 g of $ZrOCl_2·8H_2O$ was dissolved in 40 mL of water containing 10 mL of acetic acid. The resulting Zr^{4+} solution was then heated to 80 °C for 3 hours to undergo hydrolysis, leading to the formation of Zr_6O_8 clusters. After hydrolysis, the solution was diluted to a final volume of 500 mL, resulting in a 16 mM Zr^{4+} solution. Separately, 1.49 g of $BDC-NH_2$ and 0.64 g of NaOH were dissolved in water to make a 500 mL solution with a concentration of 16 mM, referred to as 16 mM $BDC-NH_2$. Similarly, another solution containing 1.33 g of BDC and 0.64 g of NaOH dissolved in water to a volume of 500 mL with a concentration of 16 mM was prepared, referred to as 16 mM BDC.

For the synthesis of non-vdW 2D UiO-66-NH$_2$ film, 5 mL of a 2 mM Zr^{4+} solution, obtained by diluting the previously prepared 16 mM Zr^{4+} solution, was mixed with a certain amount of acetic acid to adjust the final pH value in a petri dish. Subsequently, 5 mL of a 2 mM $BDC-NH_2$ solution, prepared by diluting the previously prepared 16 mM $BDC-NH_2$, was added to the petri dish at room temperature to achieve a final concentration of 1 mM for both $BDC-NH_2$ and Zr^{4+}.

Similarly, for the synthesis of non-vdW 2D UiO-66 film, 5 mL of a 1 mM Zr^{4+} solution obtained by diluting the previously prepared 16 mM Zr^{4+} solution was mixed with a certain amount of acetic acid to adjust the final pH value in a petri dish. Then, 5 mL of a 1 mM BDC solution, prepared by diluting the previously prepared 16 mM BDC solution, was added to the petri dish at room temperature to achieve a final concentration of 0.5 mM for both BDC and Zr^{4+}. The mixed solution was gently shaken for 10 seconds before placing the substrates into the solution. For AFM, SEM, and XPS measurements, highly oriented pyrolytic graphite (HOPG, ZYB quality, mosaic spread 0.6-1.0 degrees, ScanSens) was partially immersed in the synthesis solution. For synchrotron measurements, graphene/Si/SiO_2 substrates (300 nm SiO_2 coating, thickness 525±25 µm, MicroChemicals), sapphire (PI-KEM Limited), and graphene/support film for ion separation studies, were floated on the solution with the graphene or top side facing downwards (see Figure 4a). Detailed preparation procedures for these substrates are outlined in subsequent sections. After a specified duration, the substrates were removed from the solution and dried by blowing air over it. This process was repeated 1-4 times for samples designated for synchrotron measurements. All films were rinsed with deionized water after removal from the synthesis solution to eliminate residual metal salts and unreacted ligands.

- **Synthesis of non-vdW UiO-66-NH_2 membrane for ion separation**

Single-layer graphene was synthesized using low-pressure chemical vapor deposition (CVD) on a copper foil (50 mm, 99.9% purity, Strem) following established protocols.[2] The copper foil was annealed at 1077 °C in a H_2/Ar atmosphere for 60 min. To eliminate contaminants, CO_2 (100 ml min^{-1}) and H_2 (8 ml min^{-1}) were sequentially introduced for 30 min each. Subsequently, CH_4 (24 ml min^{-1}) and H_2 (8 ml min^{-1}) were introduced for 30 min to grow single-layer graphene on the copper at a pressure of 460 mtorr.

An O_2 plasma treatment (MTI Plasma Cleaner, EQ-PCE-3, 13.56 MHz, 17 W) was employed to introduce nanopores on the single-layer graphene prior to the growth of non-vdW UiO-66-NH_2 film. The plasma chamber was purged with O_2 flow until a pressure of ~50 mtorr was reached, followed by a 5.6 s plasma treatment to etch the single-layer graphene and obtain nanoporous graphene (Figure S35, Supplementary note 12).

Nanoporous graphene on copper was further reinforced with a nanoporous carbon (NPC) layer. To prepare the precursor solution, 0.4 g of PS-b-P4VP and 0.8 g of turanose were dissolved

in 4 g of DMF,[3] followed by sonication for 1 hour. The solution was then heated to 180 °C for 3 hours to obtain the NPC precursor. This precursor was spin-coated onto nanoporous graphene at 1000 rpm for 1 min. Subsequently, the NPC-coated nanoporous graphene underwent pyrolysis at 500 °C in a H_2/Ar atmosphere for 1 h, followed by cooling down at a rate of 1 °C/min to form the NPC layer. Finally, the NPC/nanoporous graphene was spin-coated with a Nafion solution and cross-linked at 120 °C for 1 h to obtain the Nafion/NPC/nanoporous graphene.

The copper foil was etched using a sequence of 1 M $FeCl_3$, 1 M HCl, and triple rinsing with deionized water. The resulting floating Nafion/NPC/nanoporous graphene films were transferred onto the surface of a premixed aqueous solution containing 1 mM Zr^{4+} and 1 mM BDC-NH_2, with the graphene side facing downwards. After a designated reaction time, the Nafion/NPC/ nanoporous graphene/UiO-66-NH_2 film was scooped onto a polytetrafluoroethylene (PTFE) support and placed between two PTFE annular disks with an 8 mm hole diameter.

- **Synthesis of non-vdW 2D UiO-66-NH_2 film on graphite nanoplatelets**

For the synthesis of non-vdW 2D UiO-66-NH_2 film on graphite nanoplatelets, nanoplatelets were initially reduced by heating at 1000 °C in a pure H_2 atmosphere for 60 minutes to remove surface contaminants. Following this, 0.5 g of the graphite nanoplatelets was introduced into the precursor solution that was same to the synthesis of non-vdW 2D UiO-66-NH_2 on graphene. The synthesis time was 30 minutes at room temperature. The resulting material was collected by centrifugation at 10000 rpm for 2 minutes. This process was repeated for a total of 10 growth cycles. After completion of the cycles, the material was washed three times with deionized water and dried overnight in a vacuum oven at 80 °C. The dried material was then ground into a fine powder and characterized using XRD, nitrogen adsorption, and TGA.

- **Non-vdW 2D UiO-66-NH_2 film on SiO_2/Si wafer using polymethyl methacrylate (PMMA) transfer**

To assess the uniformity of the non-vdW UiO-66-NH_2 film, we transferred it onto a SiO_2/Si wafer to identify regions with cracks. Initially, graphene on copper was coated with PMMA for reinforcement. Afterward, the copper was etched using a 10 wt% $Na_2S_2O_8$ solution, and the PMMA-reinforced graphene was floated on the precursor solution for film growth. The non-vdW 2D UiO-66-NH_2 film was synthesized using the same method as that for the ion-ion separation

test and then transferred onto the SiO_2/Si wafer. The PMMA layer was removed by dissolving it in acetone overnight, with the solvent refreshed three times. This process introduced cracks in the film, allowing for AFM analysis to measure the film thickness at the edges of the cracks.

- **Ion Diffusion Tests**

The ion diffusion study was conducted using a homemade diffusion cell with two 50 mL reservoirs, following our previous methodology.[3] Thin film samples were placed between two PTFE annular gaskets (effective area of ~50 mm^2) and positioned at the center of the reservoirs. The side with the MOF faced the feed reservoir containing the salt solution, while the permeate side contained Milli-Q water. During the experiment, changes in the permeate concentration were minimal compared to the feed, ensuring a constant diffusion driving force. Ion conductivity on the permeate side was monitored over time using a conductivity probe (Mettler-Toledo GmbH, SevenCompact Cond. Meter S230). Both reservoirs were continuously stirred to reduce concentration polarization. Data were validated by inductively coupled plasma-optical emission spectrometry (ICP-OES, Agilent 5110), which correlated changes in solution conductivity (μS/cm) with ion concentration (mol/L) (Figure S36) for single-ion measurements. For mixed-ion studies, each cation was analyzed over time by ICP-OES.

Electrical measurements were performed with an electrochemical workstation (Gamry) using the same H-type diffusion cell. The membrane was placed between the two chambers of the cell, each filled with a specific concentration of salt solution. Homemade Ag/AgCl electrodes were positioned in the chambers. Ionic conductance was measured across a voltage range of -0.3 to 0.3 V, with a step size of 10 mV/s, and the resulting current was recorded as a function of the applied voltage.

- **Material Characterization**

High-resolution XRD was performed at the Swiss-Norwegian Beamline BM01 at the European Synchrotron Radiation Facility (ESRF) in Grenoble, France, using a synchrotron radiation source with an X-ray wavelength of 1.04157 Å.[4, 5] The sample preparation involved coating CVD-synthesized single-layer graphene/copper with PMMA or paraffin, etching with a 10 wt% $Na_2S_2O_8$ solution, and washing with water. The coated samples were then transferred to a clean SiO_2/Si wafer. PMMA or paraffin was removed by washing with acetone or heptane, respectively at least three times with fresh solvents to remove the polymer. The graphene/SiO_2/Si substrate was cleaned

under H_2 flow at 900 °C for 3 hours, followed by floating on the synthesis solution for non-vdW 2D UiO-66-NH_2 or UiO-66 film growth. The detailed operation procedure is stated in Supplementary note 5 and the measurement geometry is shown in Figure 3a. In brief, the thin film sample was mounted horizontally and shaded by half of the beam. The optimal condition was determined by rotating the sample to gain the highest intensity from the sample.

XPS measurements were conducted on UiO-66-NH_2/HOPG using an Axis Supra instrument from Kratos Analytical, with a monochromated Kα X-ray source emitted from an aluminum anode. Parameters were set at a pass energy of 40 eV and a step size of 0.1 eV. The samples were grounded to prevent charge accumulation. The binding energy data were utilized without adjustments, and CasaXPS software was used for the data processing, including background subtraction using the Shirley method.

Raman spectroscopy was performed using a Renishaw inVia InSpect Raman confocal microscope with a 457 nm excitation laser. The laser power was maintained at below 1 mW to prevent sample damage. I_D/I_G ratios were determined by subtracting background signals and using curve fitting techniques to identify G and D peaks, analyzed with Origin software.

SEM measurements were conducted using SEM Teneo (FEI) operating at 1 kV at a working distance of 8-10 mm.

Powder X-ray diffraction was measured using a Bruker D8 Advance diffractometer with a Lynxeye XE detector. The instrument operated at 40 kV and 40 mA with Cu Kα radiation (λ = 1.5406 Å), and measurements were conducted at ambient temperature and pressure.

AFM images were obtained using a Bruker MultiMode 8 AFM with a SCANASYST-AIR probe, featuring a tip radius of 2 nm, a frequency of 70 ± 25 kHz, and a spring constant of approximately 0.4 N/m. The surface roughness (RMS) was analyzed across three distinct regions using Gwyddion software.

Modulus measurement was used by a Bruker Tap525A rectangular probe, operating in PeakForce QNM in Air mode. Prior to measurement, the probe was calibrated using standard samples of sapphire, polystyrene, and PDMS to update the probe sensitivity, spring constant (K), and tip

radius. After calibration, the probe sensitivity, spring constant, and tip radius were determined to be 60.02 nm/V, 0.1228 N/m, and 28.66 nm, respectively.

AC-HRTEM was performed with a double-corrected Titan Themis 60-300 transmission electron microscope (FEI, Thermo Fisher Scientific) equipped with a Wein-type monochromator. Single-layer graphene, transferred onto UltrAuFoil® with 0.6 µm holes and initially reinforced with paraffin (which was later removed using heptane), was cleaned at 300 °C in a CO_2 atmosphere for 12 hours to reduce atmospheric contamination. Samples were then subjected to a 5.6-second plasma treatment to simulate membrane fabrication conditions. Imaging was carried out at 80 kV with a monochromated electron beam to reduce knock-on effects and chromatic aberration. A negative spherical aberration (C_s) of ~18 µm was used to enhance imaging quality. SAED was performed using a Thermo Fisher Scientific Spectra200 at 200 kV.

TGA measurement was performed to quantify the loading of non-vdW 2D UiO-66-NH_2 film grown on graphite nanoplatelets. The analysis was carried out using a PerkinElmer Thermogravimetric Analyzer TGA8000 with an air flow rate of 20 mL/min. Before the temperature ramp, all samples were subjected to vacuum at 80 °C overnight and then cooled to room temperature for activation. The temperature was then ramped at a rate of 5 °C/min from 30 °C to 800 °C.

N_2 adsorption measurements were carried out on graphite nanoplatelets and non-vdW 2D UiO-66-NH_2 films deposited on graphite. The samples were transferred into pre-weighed glass sample tubes under a nitrogen atmosphere and sealed. They were then moved to the activation station (Belsorp Vac II), where they were placed under vacuum at room temperature for 24 hours to ensure complete evacuation. Activation was considered complete when the outgas rate at 523 K was less than 2 µbar/min. After activation, the tubes containing degassed samples were weighed to determine the sample mass. Subsequently, the tubes were then transferred to the analysis port of the Belsorp Max instrument to measure nitrogen gas adsorption isotherms at 77 K using liquid nitrogen.

Table S1 Thickness comparison of our films compared to the state-of-the-art membranes in the literatures.

No.	Thickness (nm)	Functional group	Ref.
	**1.7 ± 0.2**	**NH_2**	**This work**
	**3.8 ± 0.4**	**NH_2**	
	**4.7 ± 0.4**	**NH_2**	
1	100 ± 10	H	6
2	103 ± 14	H	7
3	500-1000	H	8
4	1000	NH_2	9
5	~1000	H (with crown ether)	10
6	1200	H	11
7	1200-1500	H	12
8	2000	H	13
9	~2900	H	14
10	3000	NH_2	15
11	4000	NH_2	16
12	5000	H	17
13	5000	H	18
14	5700	NH_2	19
15	8000	NH_2	20
16	12000 (nanochannel)	H	21
17	15000	H	22

Table S2 Different synthesis conditions and the corresponding pH value by adding different amounts of acetic acid.

**Zr^{4+} (mM)**	0.5	1	0.5	0.5	0.5	2	2	2	1	1	1	**1**
**Ligand (mM)**	4	8	5	5	5	2	2	2	1	1	1	**1**
**pH**	4.77	4.67	4.84	3.89	3.63	3.49	3.44	3.27	3.69	3.51	3.40	**3.30**

Table S3 Comparison of reaction time, temperature, and solvent used in this study and those reported in the literatures.

Solvent	Synthesis temperature (°C)	Synthesis time (min)	Functional group	Ref.
**water**	**25**	**5**	**NH_2**	
**water**	**25**	**20**	**NH_2**	**This work**
**water**	**25**	**30**	**NH_2**	
DMF	100	720	H	[12]
DMF	80	180	H	[14]
DMF	180	1440	H	[17]
DMF	120	1440	H	[18]
DMF	120	1440	H	[8]
DMF	120	4320	H	[13]
DMF	120	1440	H	[22]
DMF	120	1440	NH_2	[15]
DMF	140	2880	NH_2	[19]
DMF	120	2880	NH_2	[16]
DMF	120	1440	NH_2	[9]
DMF	150	2880	NH_2	[20]
DMF	80	720 (overnight)	H	[23]
DMF	100	1440	H	[21]
DMF	220	960	H	[6]
DMF	120	4320	H	[11]
DMF	120	2880	H	[7]

Table S4 Comparison of Zr^{4+} and BDC-x concentration used in this study and those reported in the literature.

Zr^{4+} (mM)	Ligand (mM)	Zr^{4+} (wt%)	Ligand (wt%)	Functional group	Ref
**1.00**	**1.00**	**0.01**	**0.02**	**NH_2**	**This work**
30.00	30.00	0.27	0.54	H	[12]
14.00	19.75	0.13	0.36	H	[14]
82.86	174.35	0.76	3.16	H	[17]
9.00	9.00	0.08	0.16	H	[18]
11.54	11.54	0.11	0.21	H	[8]
9.43	9.43	0.09	0.17	H	[8]
6.91	6.91	0.06	0.13	H	[8]
25.82	25.82	0.24	0.47	H	[13]
100.00	25.00	0.91	0.45	H	[22]
100.00	40.00	0.91	0.72	H	[22]
100.00	50.00	0.91	0.91	H	[22]
100.00	84.00	0.91	1.52	H	[22]
100.00	70.00	0.91	1.27	H	[22]
100.00	60.00	0.91	1.09	H	[22]
17.15	17.15	0.16	0.31	NH_2	[15]
21.14	21.14	0.19	0.38	NH_2	[19]
21.14	21.14	0.19	0.38	NH_2	[16]
25.82	25.82	0.24	0.47	NH_2	[9]
41.67	41.67	0.38	0.75	NH_2	[20]
33.75	46.88	0.31	0.85	H	[23]
25.83	25.83	0.24	0.47	H	[21]
25.83	25.83	0.24	0.47	H	[6]
25.09	25.09	0.23	0.45	H	[6]
25.82	25.82	0.24	0.47	H	[11]
25.82	77.46	0.24	1.40	H	[11]
25.82	129.10	0.24	2.34	H	[11]

25.82	180.73	0.24	3.27	H	[11]
25.79	25.79	0.24	0.47	H	[7]
25.27	25.27	0.23	0.46	H	[7]
24.91	24.91	0.23	0.45	H	[7]
24.05	24.05	0.22	0.44	H	[7]
23.25	23.25	0.21	0.42	H	[7]

Table S5 Comparison of the defective ratio for UiO-66-NH_2 powder and non-vdW 2D UiO-66-NH_2 film using TGA.

Sample	defective ratio by TGA
UiO-66-NH_2 powder	0.32
Non-vdW 2D UiO-66-NH_2	0.30

***Note:** The TGA analyses reveal a defect ratio of 0.32 for UiO-66-NH_2 powder and 0.30 for the non-vdW UiO-66-NH_2 film. This confirms that the non-vdW UiO-66-NH_2 film possesses a significant level of defects. Besides, the slightly lower defect ratio in the film compared to the powder facilitates its synthesis and improves its stability.

Table S6 The relation of incidence angle and measurement footprint.

Incidence angle (°)	Footprint (cm)
0.08	2.65
0.09	2.36
0.10	2.12
0.11	1.93
0.12	1.77
0.13	1.63
0.14	1.51

Table S7 The ion flux of non-vdW 2D UiO-66-NH_2, UiO-66, and porous graphene.

	Ion flux (mol m^{-2} h^{-1})			
	K^+	Na^+	Li^+	Mg^{2+}
Non-vdW 2D UiO-66-NH_2	1.54×10^{-3}	5.74×10^{-4}	6.96×10^{-4}	1.54×10^{-5}
	5.25×10^{-4}	2.85×10^{-4}	3.50×10^{-4}	3.01×10^{-5}
	2.67×10^{-3}	7.45×10^{-4}	8.10×10^{-4}	3.21×10^{-5}
Non-vdW 2D UiO-66	3.10×10^{-3}	1.79×10^{-3}	4.95×10^{-3}	3.12×10^{-4}
	2.70×10^{-3}	1.85×10^{-3}	4.82×10^{-3}	2.31×10^{-4}
	4.61×10^{-3}	1.93×10^{-3}	3.05×10^{-3}	3.40×10^{-4}
Porous graphene	5.55×10^{-2}	2.58×10^{-2}	4.46×10^{-2}	1.92×10^{-2}
	3.26×10^{-2}	2.51×10^{-2}	3.08×10^{-2}	1.11×10^{-2}
	4.80×10^{-2}	3.17×10^{-2}	4.21×10^{-2}	1.52×10^{-2}

Table S8 Comparison of ion selectivity and flux with other materials.

Membrane type	K^+/Mg^{2+} selectivity	K^+ flux (mol m^{-2} h^{-1})	Thickness (nm)	Ref.
Nafion 212	2.44	1.53×10^{0}	300	[24]
i-CMP	40.9	7.67×10^{-1}	40	[25]
CMP	7.07	1.01×10^{-1}	41.3	[25]
CMP	1.67	1.05×10^{-1}	300	[26]
PA	2	6.00×10^{-3}	15	[27]
PIM	12.3	1.08×10^{1}	300	[24]
PIM	31.3	1.22×10^{0}	300	[24]
PIM	48.8	2.00×10^{-1}	300	[24]
PIM	42.1	1.60×10^{0}	300	[24]
PIM	3.01	2.40×10^{0}	300	[24]
MXene	5.88	9.40×10^{-1}	1500	[28]
MXene	4.4	2.20×10^{-1}	50	[29]
MXene	2.8	7.00×10^{-2}	80	[29]
MXene	1.39	3.78×10^{-1}	300	[30]
MXene	4.62	2.54×10^{-2}	300	[30]
GO	9.7	5.50×10^{-2}	200	[31]
GO	50	1.90×10^{-3}	200	[31]
GO	11	3.60×10^{-3}	200	[31]
GO	90.3	8.90×10^{-4}	200	[31]
GO	3.69	4.95×10^{-1}	~500	[32]
GO	9.11	1.36×10^{0}	~500	[32]
Al-MOF	1.06	6.88×10^{-6}	100	[33]
**Non-vdW 2D UiO-66-NH_2**	**99.8**	**1.54×10^{-3}**	**~5**	**This work**
	**83.2**	**2.67×10^{-3}**		

Table S9 Comparison of the non-vdW 2D $UiO\text{-}66\text{-}NH_2$ film with other reported oriented MOF film.

MOFs	Thickness (nm)	Dense structure	Ref.
ZIF-8	150	Yes	[34]
$UiO\text{-}66\text{-}NH_2$	235	No	[35]
UiO-67	440	No	[35]
PPPP-PIZOF-1	2500	No	[35]
$Fe(bpy)[Pt(CN)_4]$	~30	No	[36]
$Cu_2(BDC)_2$	> 1000	No	[37]
**non-vdW 2D $UiO\text{-}66\text{-}NH_2$**	**~2-5**	**Yes**	**This work**

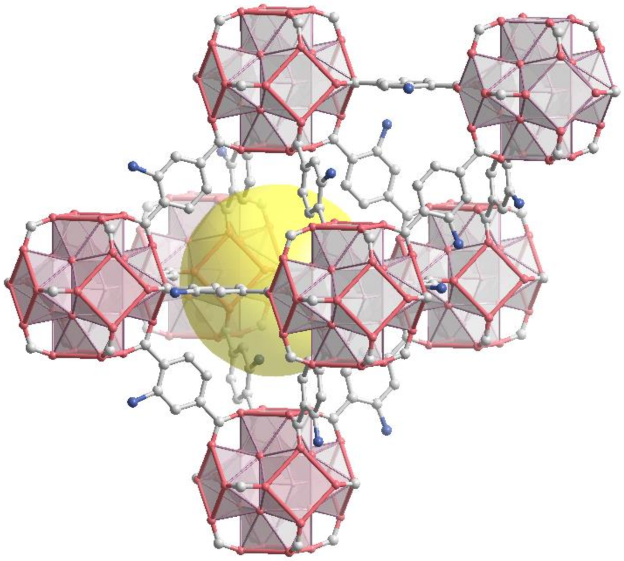

Figure S1 Schematic representation of the UiO-66-NH_2 structure, demonstrating an octahedral cage sharing a triangular face with a tetrahedral cage. Nitrogen (blue), carbon (grey), zirconium (pink), and oxygen (red) atoms are shown. Hydrogen atoms are omitted for simplicity. The yellow sphere indicates the empty space within the octahedral cage.

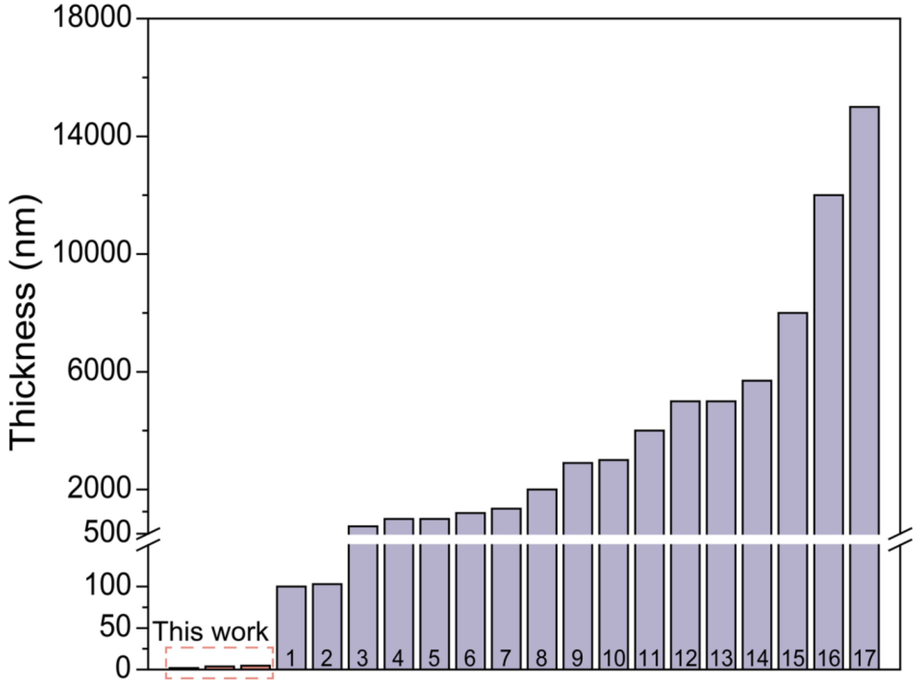

Figure S2 Comparison of the thickness of our films with state-of-the-art UiO-66 membranes reported in the literature. Statistical results and references are summarized in Table S1.

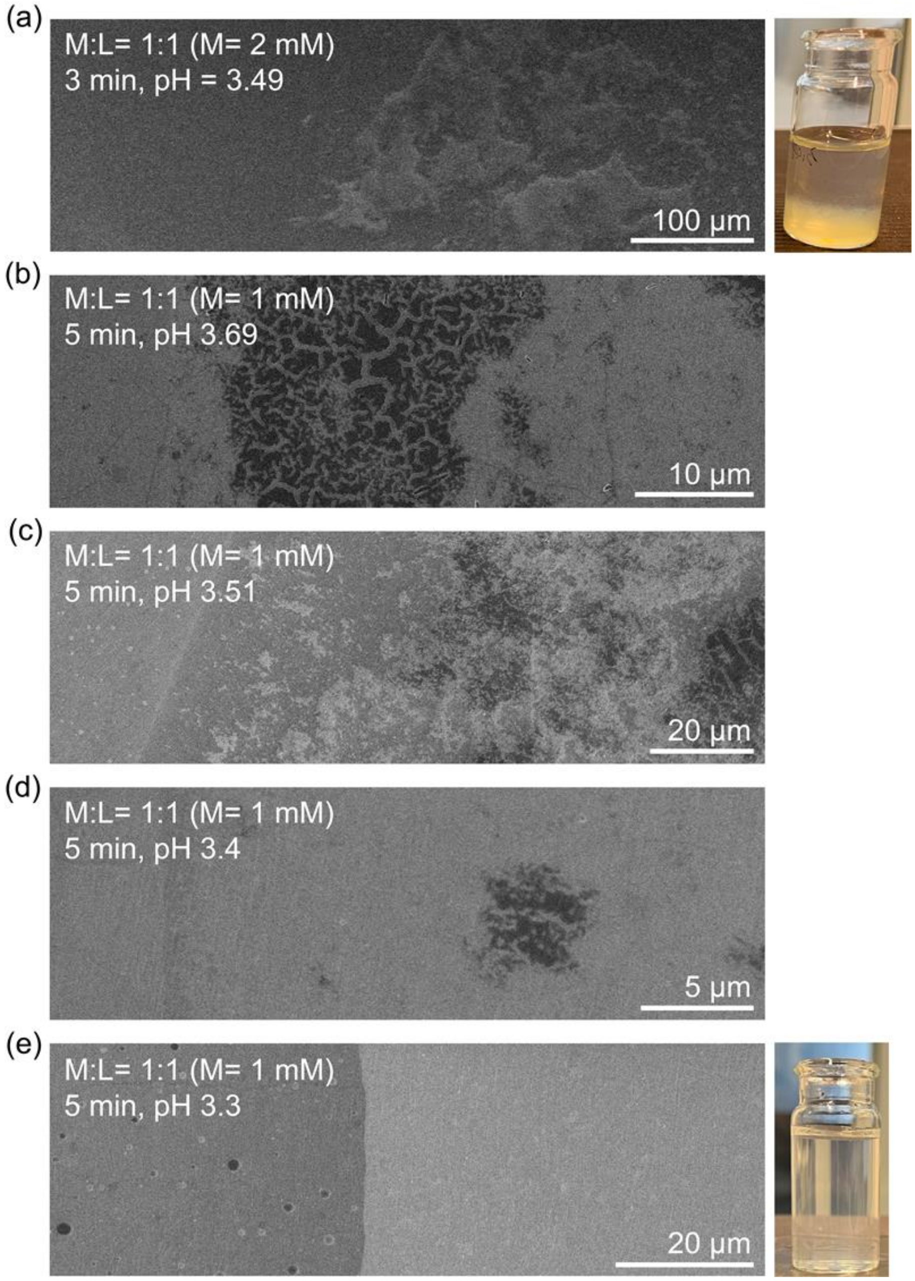

Figure S3 SEM images of different synthesis conditions with different concentrations and pH values. The photograph of the synthesis solution was taken after 10 min of reaction.

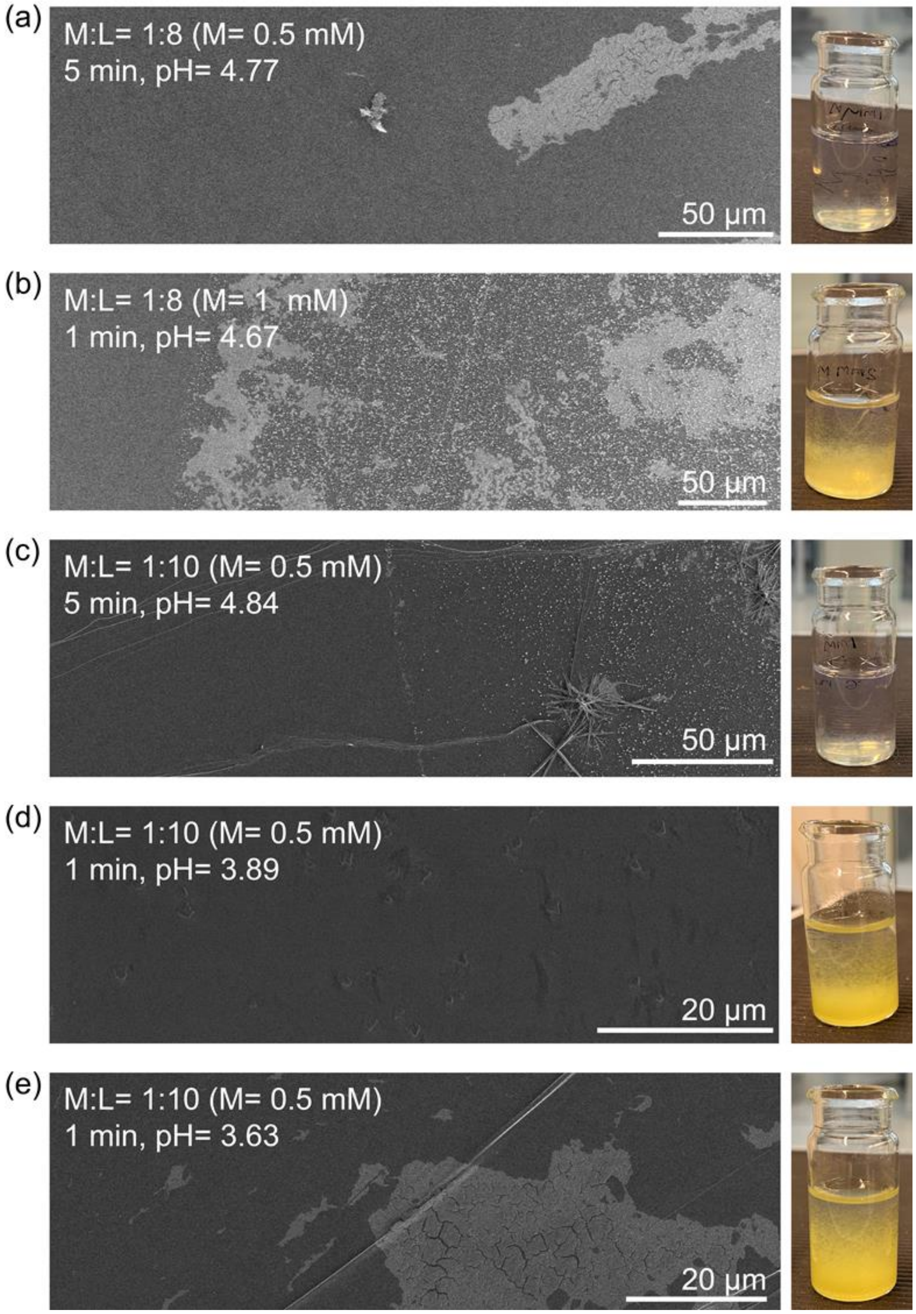

Figure S4 SEM images of different synthesis conditions with the ligand-to-metal ratio of (a, b) 8 and (c-e) 10. The photographs of the synthesis solutions were taken after 10 min of reaction.

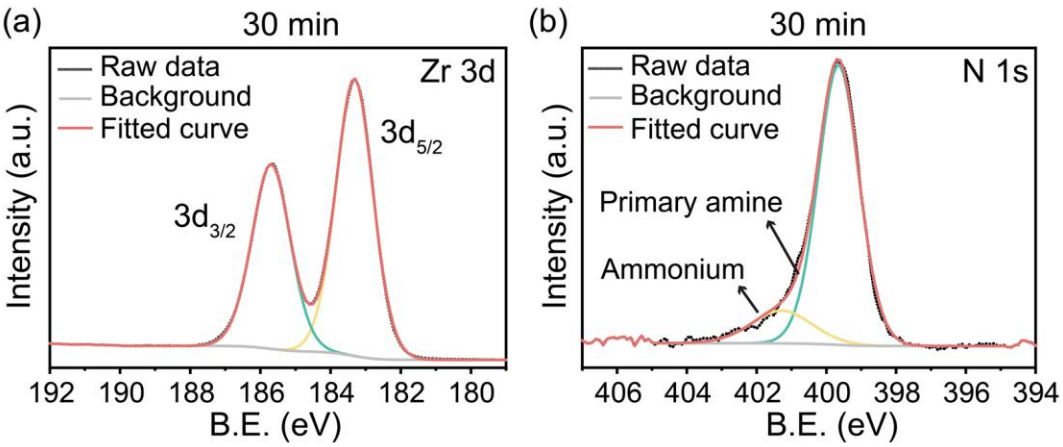

Figure S5 Zr 3d and N 1s XPS spectra from non-vdW 2D UiO-66-NH_2 films with a growth time of 30 min.

Supplementary note 1: Zr 3d and N 1s XPS spectra of highly crystalline UiO-66-NH_2 powder

A high-crystalline UiO-66-NH_2 powder, prepared using aqueous synthesis at 100 °C for 12 hours for comparative analysis, had its crystal structure verified via X-ray diffraction (XRD) (Figure S6a). The BE for Zr 3d and N 1s (Figure S6c and d) are consistent with the literatures,[24, 25] and those observed in our thin films (Figure 1f-g, S5). The powder exhibited a lower N/Zr ratio of 0.47, suggesting a higher linker vacancy defect density of ~53% compared to the thin films (Figure S6b). This demonstrates that high crystalline UiO-66-NH_2 synthesized in aqueous solution can also have a high defect density.

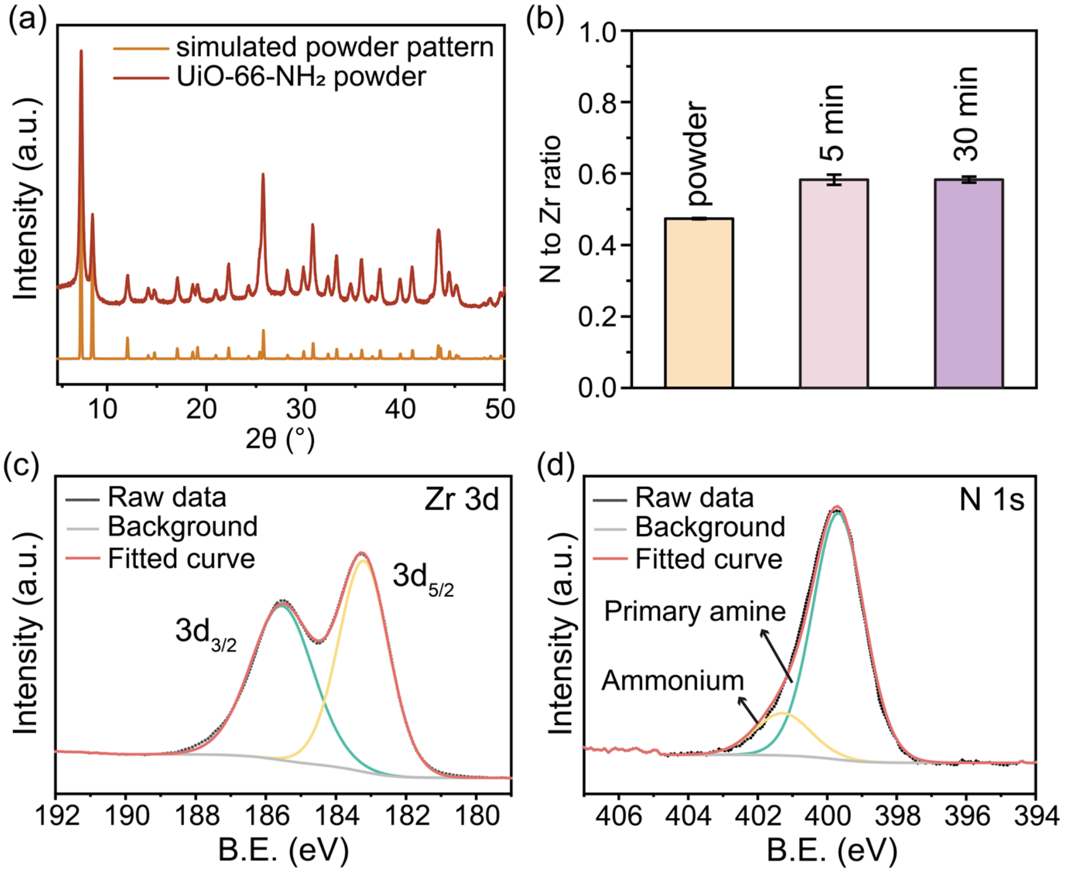

Figure S6 (a) XRD patterns of powder sample. (b) Comparison of the N/Zr ratio between non-vdW 2D UiO-66-NH_2 films and the powder sample. Error bars represent the standard deviation of XPS measurements taken from three different regions. (c) Zr 3d and (d) N 1s XPS spectra of powder sample.

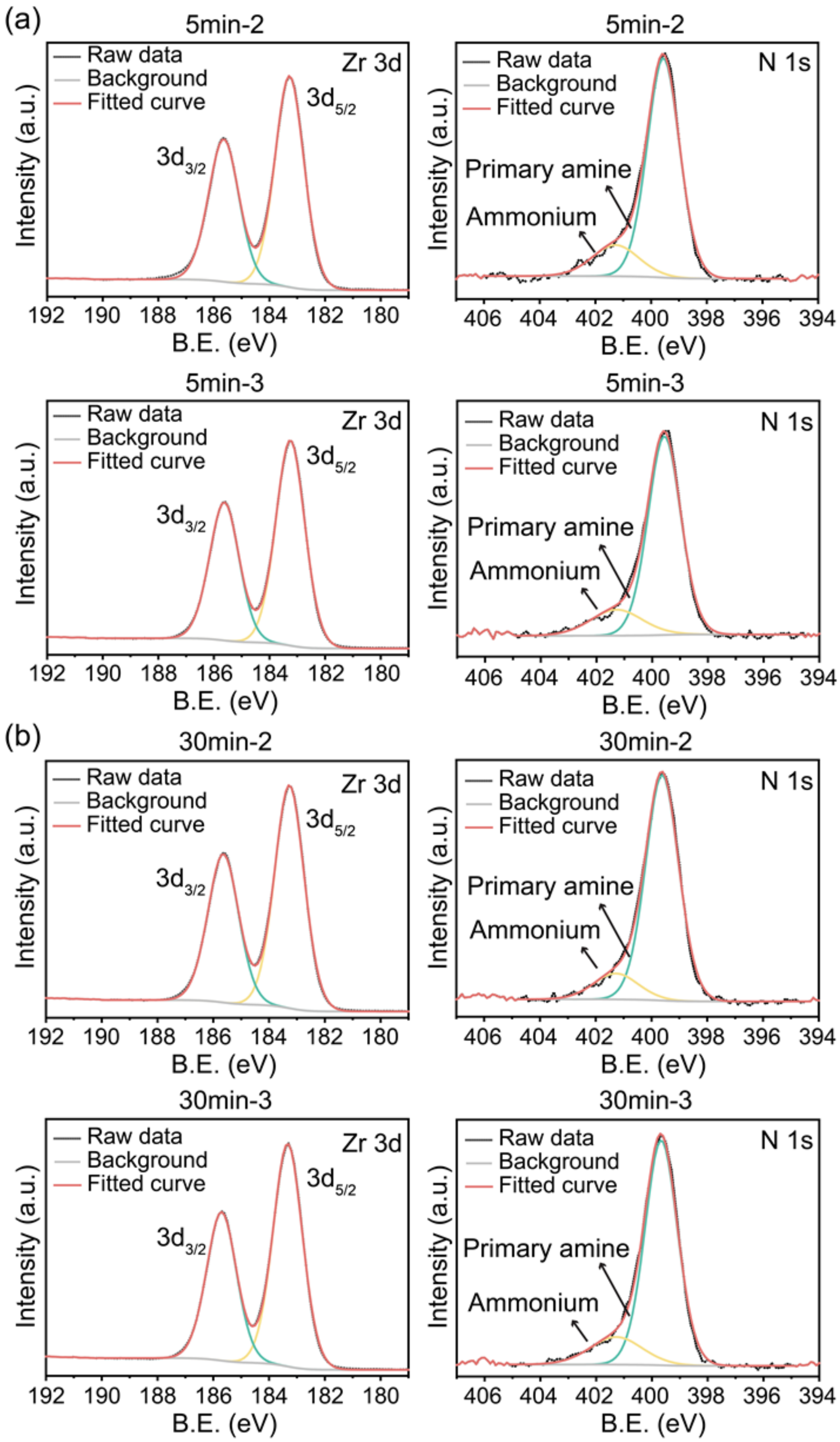

Figure S7 Zr 3d and N 1s XPS spectra from non-vdW 2D UiO-66-NH_2 films with a growth time of (a) 5 min and (b) 30 min measured from different regions of the films. X min-2 and X min-3 refer to the second and third regions analyzed on the thin films.

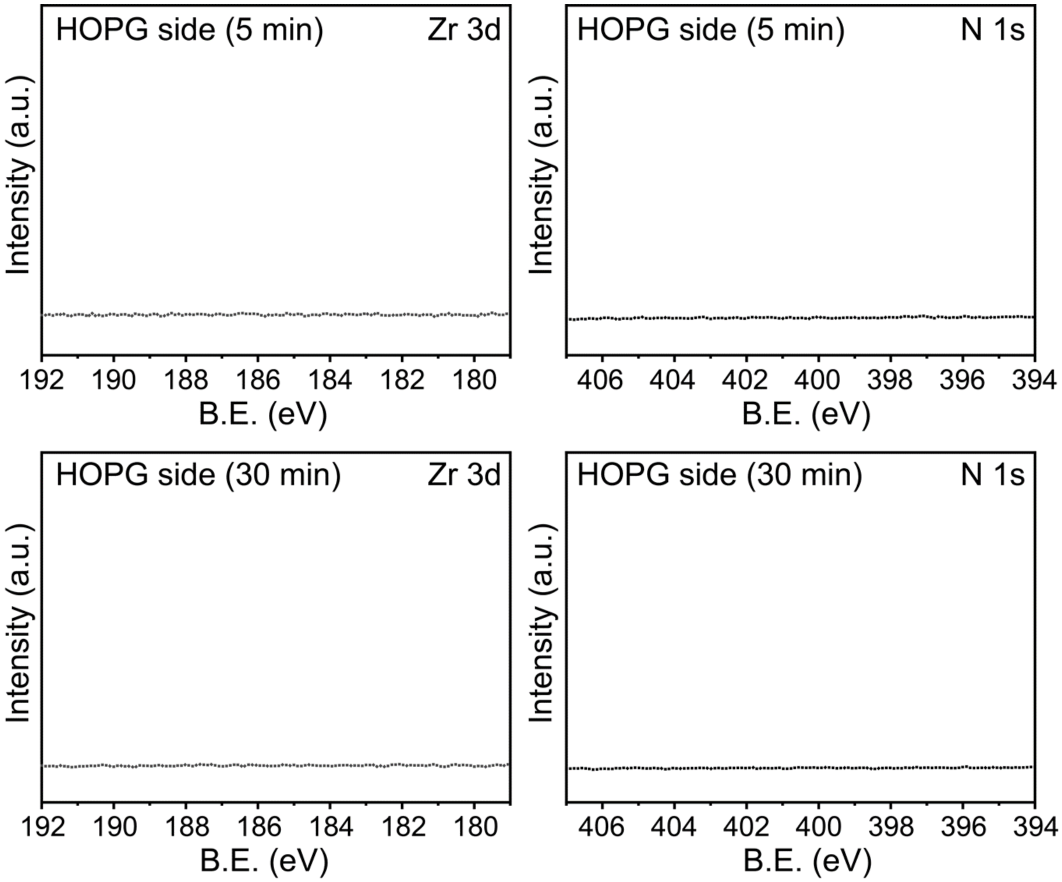

Figure S8 Zr 3d and N 1s XPS spectra from HOPG side with a growth time of 5 min and 30 min.

Supplementary note 2: Quantitative stoichiometry analysis of non-vdW 2D UiO-66-NH_2 films using XPS

The stoichiometry of N to Zr in UiO-66-NH_2 films was evaluated through XPS analysis, focusing on data collected from three distinct areas of the films. Quantification is based on the intensity of Zr 3d within BE range of 190.09 eV to 176.16 eV, and N 1s in the range of 404.62 eV to 395.17 eV. The approach is justified by the presence of Zr within the metal clusters and N originating from BDC-NH_2 in the structure of UiO-66-NH_2. The loss in ligand implicates a corresponding reduction in nitrogen content. Quantitative analysis was facilitated by CasaXPS software, employing relative sensitivity factors (RSFs) specific to the instrumentation to convert intensity data into stoichiometric values.[38] For accurate background correction, the Shirley background was applied. Given the ideal stoichiometric composition of UiO-66-NH_2 is represented as $Zr_6O_6(BDC\text{-}NH_2)_6$, the theoretical N:Zr ratio stands at 1:1, assuming a defect-free structure.

The quantification of defect density was determined using the formula (1):

$$defect\ density = I_{N\ 1s}/I_{Zr\ 3d} \tag{1}$$

where $I_{N\ 1s}$ and $I_{Zr\ 3d}$ denote the intensity of N 1s and Zr 3d, respectively.

Supplementary note 3: Quantitative stoichiometry analysis of UiO-66-NH_2 powder and non-vdW 2D UiO-66-NH_2 films using TGA

The mass of film growing on centimeter-scale HOPG is few hundred nanograms. This makes it extremely challenging to carry out thermogravimetric analysis (TGA) and nitrogen sorption analysis which require several milligrams of a material. To achieve a sufficient quantity, we crystallized non-vdW 2D UiO-66-NH_2 on powdered graphite nanoplatelets with thickness of 2-10 nm. The powder XRD pattern of the non-vdW 2D UiO-66-NH_2 film had characteristic UiO-66-NH_2 peaks at 111 and 200 (Figure S9), confirming that the synthesized material was crystalline.

The loading of non-vdW 2D UiO-66-NH_2 film on graphite was quantified using the following equation based on TGA results, focusing on data collected from 8-10 mg of each sample (Figure S10):

$$mass\ ratio\ (wt\%) = \frac{\Delta m_{UiO\ on\ graphite} - \Delta m_{graphite}}{\Delta m_{UiO} - \Delta m_{graphite}} \times 100\%$$

where $\Delta m_{UiO\ on\ graphite}$, $\Delta m_{graphite}$ and Δm_{UiO} are the mass losses of the non-vdW 2D UiO-66-NH_2 grown on the graphite nanoplatelets, the graphite nanoplatelets and the UiO-66-NH_2 powder in the temperature range from 30 to 450 °C, respectively. This calculation determined that the MOF loading on the graphite nanoplatelets was approximately 11.24 wt%.

The defect density for the non-vdW 2D UiO-66-NH_2 on graphite and UiO-66-NH_2 powder was determined using weight loss from the ligand and the residue from ZrO_2 (Figure S10). In the temperature range of 350-450 °C, the mass loss is mainly attribute to the removal of BDC-NH_2,[39] which constituted 37.81 wt%. The remaining residue after 450 °C, accounting for 37.91%, is attributed to ZrO_2. Using these values, we calculated the molar ratio of N to Zr to be 0.68, indicating a defect density of 32 % for the UiO-66-NH_2 powder. Similarly, the defect ratio of the non-vdW 2D UiO-66-NH_2 film was calculated to be 0.70, corresponding to a 30 % defect density, which is slightly lower than that of the powder. A comparison of the defect ratios obtained through both XPS and TGA is provided in Table S5.

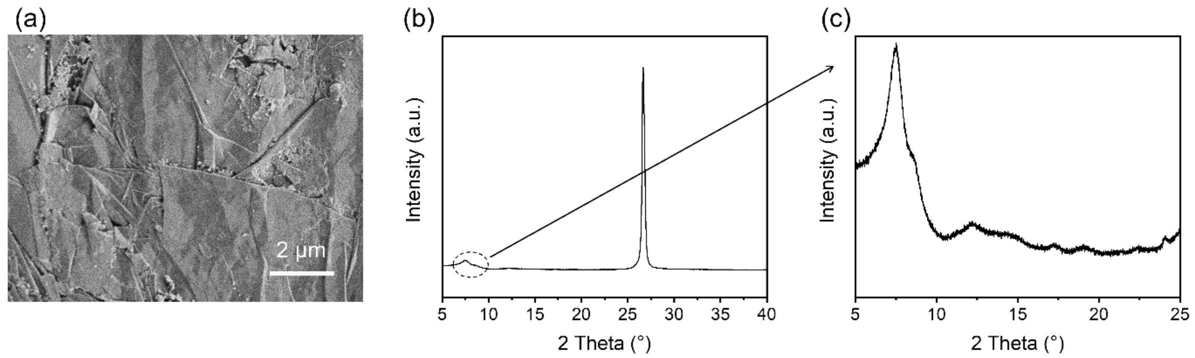

Figure S9 Morphology and crystallinity of the non-vdW 2D UiO-66-NH_2 film synthesized on graphite nanoplatelets: (a) SEM image and (b) XRD pattern, with (c) the corresponding magnified view highlighted in the dashed circle.

***Note:** We could see the resulting MOF film on graphite nanoplatelets has a smooth morphology, and no 3D grain could be observed. Besides, the powder XRD pattern of the non-vdW 2D UiO-66-NH_2 film shows characteristic peaks at 111 and 200, confirming the film identity as UiO-66-NH_2.

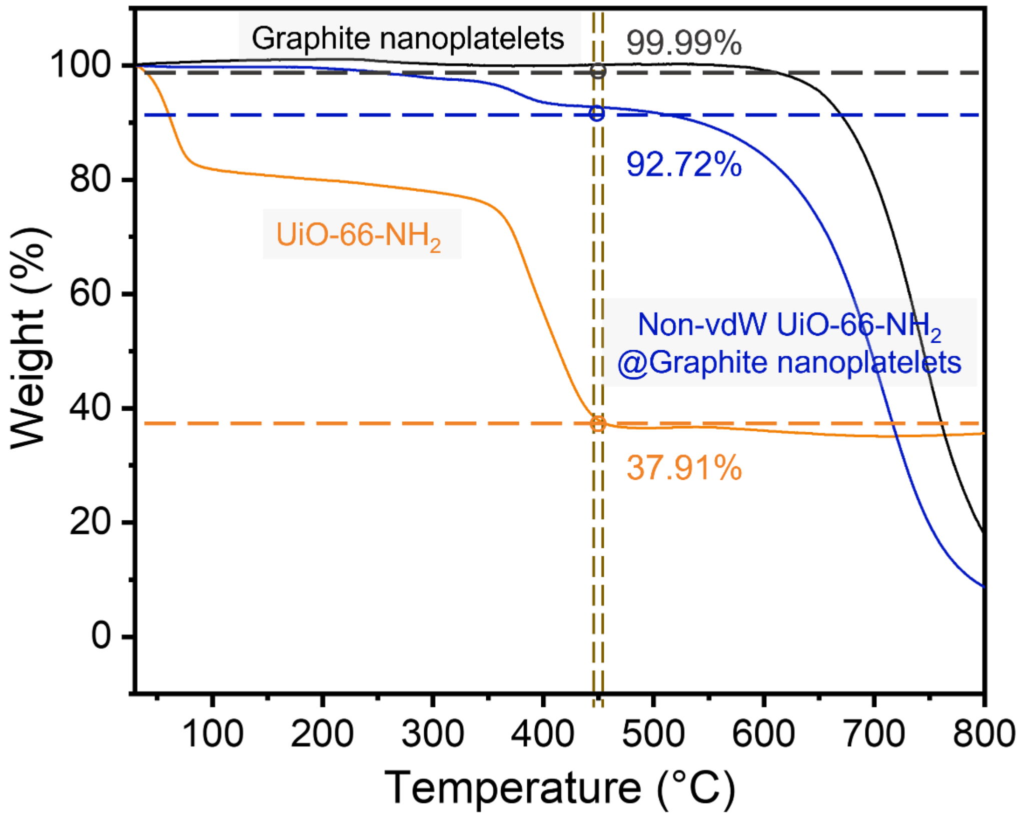

Figure S10 TGA data for UiO-66-NH_2 powder, non-vdW 2D UiO-66-NH_2, and graphite nanoplatelets.

***Note:** TGA indicated that approximately 11.24% of the total mass consisted of non-vdW 2D UiO-66-NH_2.

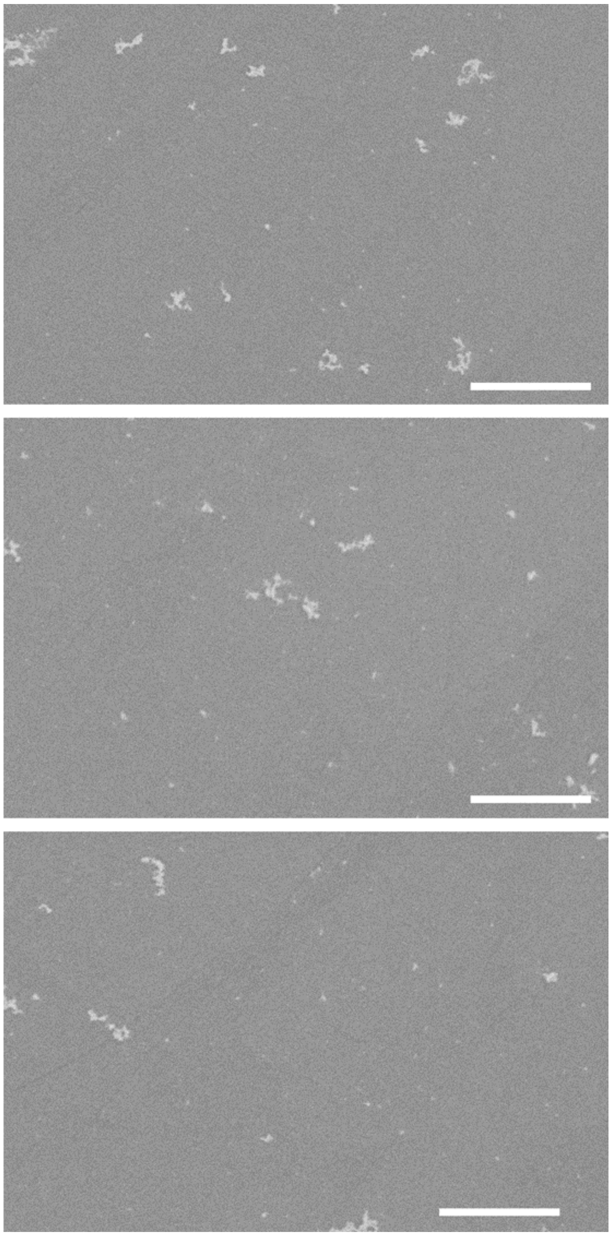

Figure S11 SEM images of non-vdW 2D UiO-66-NH_2 film synthesized for 30 min, taken from positions far from the edge, demonstrating film uniformity at different locations. Scale bar: 1 μm.

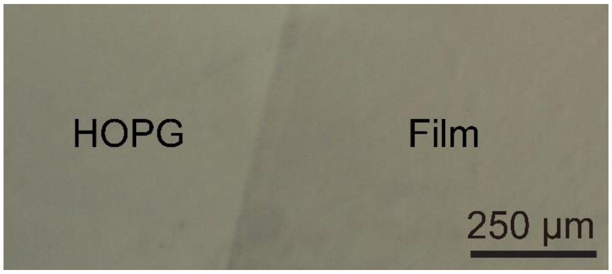

Figure S12 Optical microscopy image of non-vdW UiO-66-NH_2 film synthesized for 30 min.

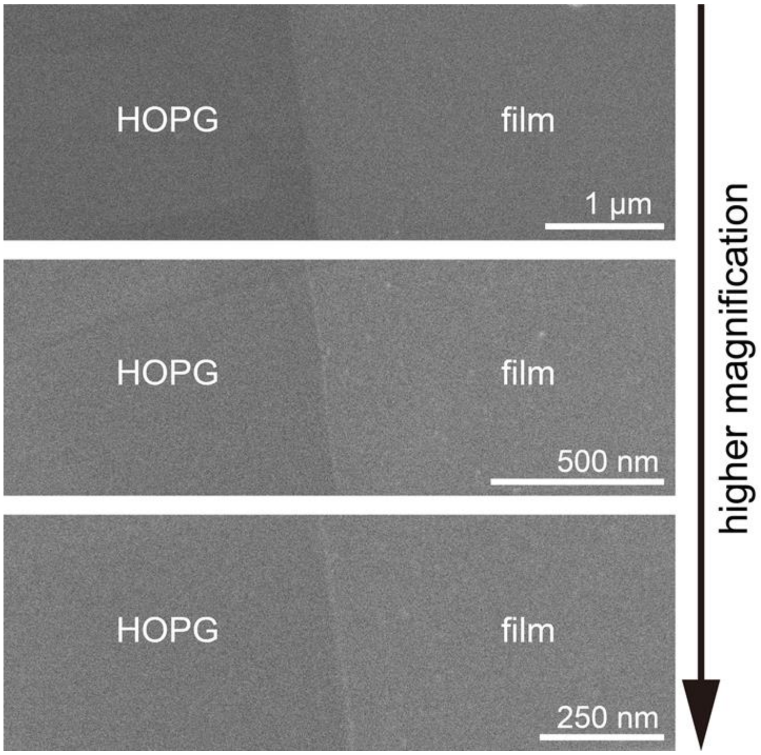

Figure S13 SEM images of non-vdW UiO-66-NH_2 film synthesized in 5 min with different magnification.

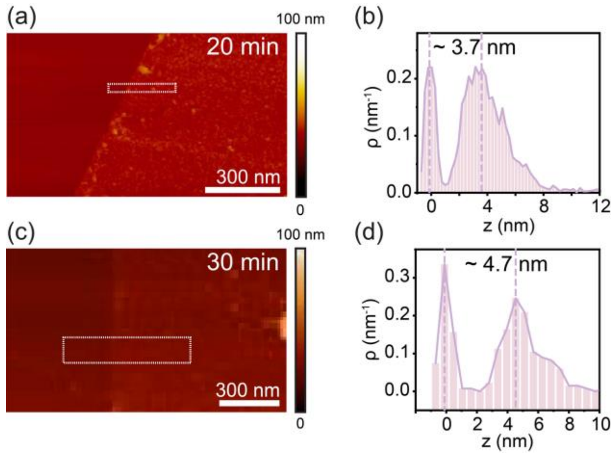

Figure S14 AFM images and the corresponding height profile of (a, b) 20 min and (c, d) 30 min growth of non-vdW 2D UiO-66-NH_2 films.

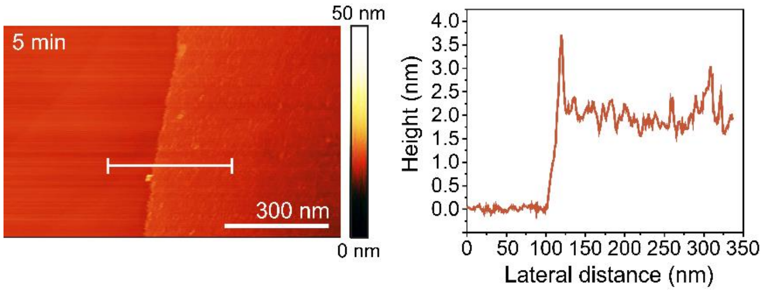

Figure S15 AFM image and corresponding height profile of non-vdW 2D UiO-66-NH_2 film synthesized in 5 min. The white line in the left panel corresponds to the lateral distance in the right panel.

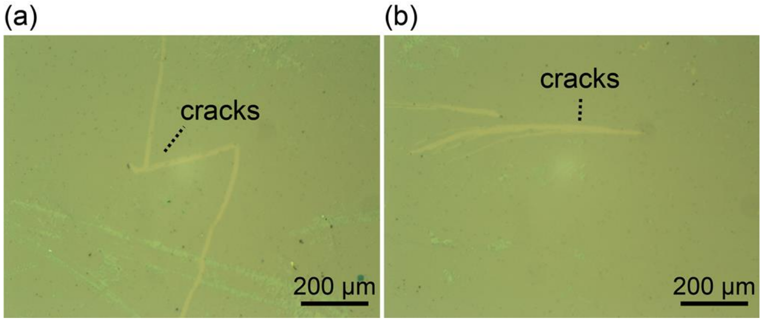

Figure S16 Optical microscopy images of the non-vdW 2D UiO-66-NH_2 film after PMMA removal. The image reveals a smooth surface with visible cracks, which allow for thickness measurement along the crack lines.

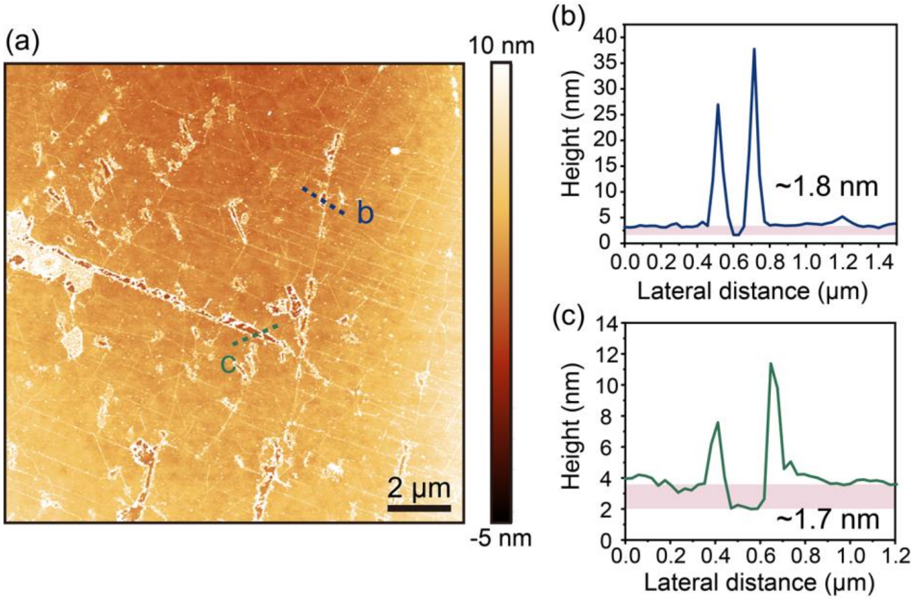

Figure S17 (a) AFM image of the transferred non-vdW 2D UiO-66-NH_2 film synthesized for 5 minutes on a SiO_2/Si wafer. (b, c) Corresponding height profiles along dashed line b and c in panel (a).

***Note:** To assess the uniformity of the non-vdW UiO-66-NH_2 film, we used AFM to measure thickness at various cracks. The AFM scan of the uncracked surface reveals a smooth layer without prominent MOF particles. Thickness measurements at cracks, specifically at positions b and c, show consistent values around 1.7-1.8 nm, matching the thickness observed at the film/HOPG interface. This confirms that the non-vdW 2D UiO-66-NH_2 film is uniformly grown on the whole substrate with the consistent thickness.

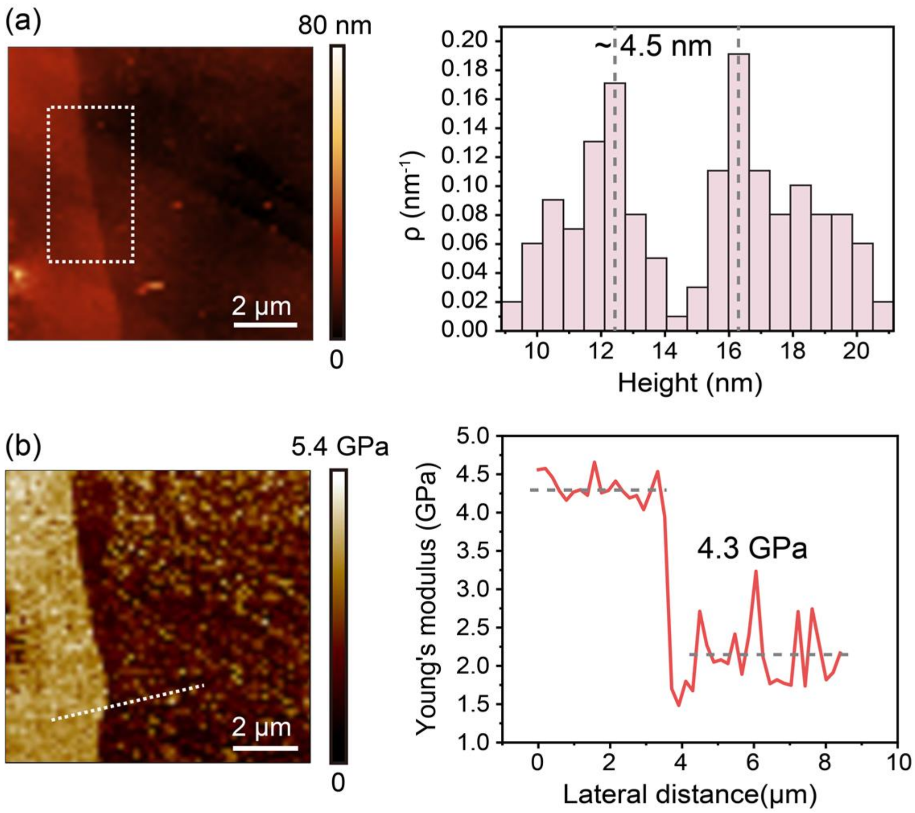

Figure S18 (a) AFM images of the UiO-66-NH_2 film grown on HOPG and the corresponding height distribution within the region outlined by the white dashed line. (b) The corresponding modulus map, and the modulus distribution along the white dashed line.

***Note:** To evaluate the mechanical strength of the non-vdW 2D UiO-66-NH_2 film, we used an AFM modulus map to quantify its properties. The non-vdW 2D UiO-66-NH_2 film synthesized over 30 minutes demonstrated a Young's modulus of 4.3 ± 0.3 GPa, indicating significant robustness for 30-minute-grown non-vdW UiO-66-NH_2 film.

Supplementary note 4: Estimation of UiO-66-NH_2 film thickness

The thickness of UiO-66-NH_2 films was determined by considering the unit cell size of UiO-66-NH_2, which is 2.074 nm (Figure S19).[40] This unit cell dimension accounts for a mid-section cut through the metal cluster, serving as a foundation for thickness calculation. Our analysis focused on the structural orientation along the 200 direction, which was confirmed by GIWAXS results.

Building upon insights from the previous study, which revealed that 100 surfaces are mostly terminated with metal clusters,[41] our thickness estimations were calculated by the complete molecular structure of UiO-66-NH_2, featuring entire metal clusters at both the lower and upper ends of the framework. Contrasting with traditional assessments of a half unit cell, typically bisected through the metal cluster to yield a measurement of 1.037 nm, our analysis considered the full presence of the metal clusters. As illustrated in Figure 2e, our analysis yielded the following thickness measurements: 1.68 nm for 0.5 unit cell (Figure S19), 3.68 nm for 1.5 unit cells, and 4.68 nm for two unit cells, considering the entirety of the metal clusters, thereby offering a more accurate representation of the films' thickness.

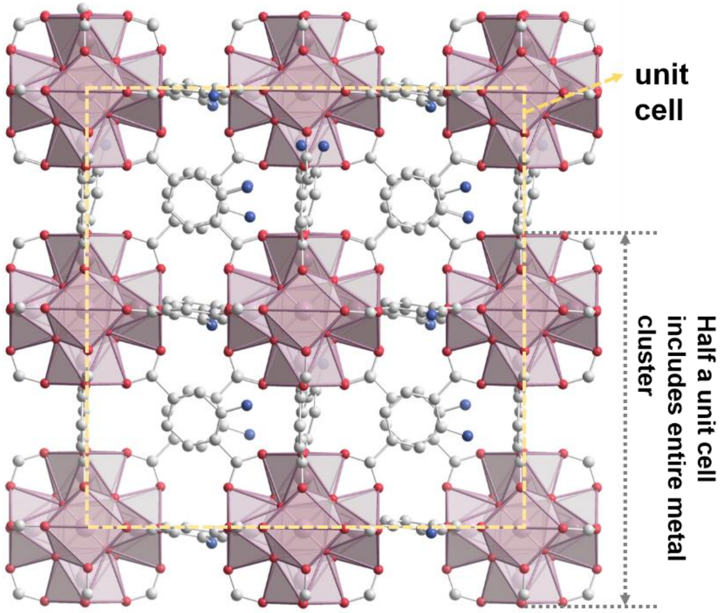

Figure S19 The molecular structure of UiO-66-NH_2.

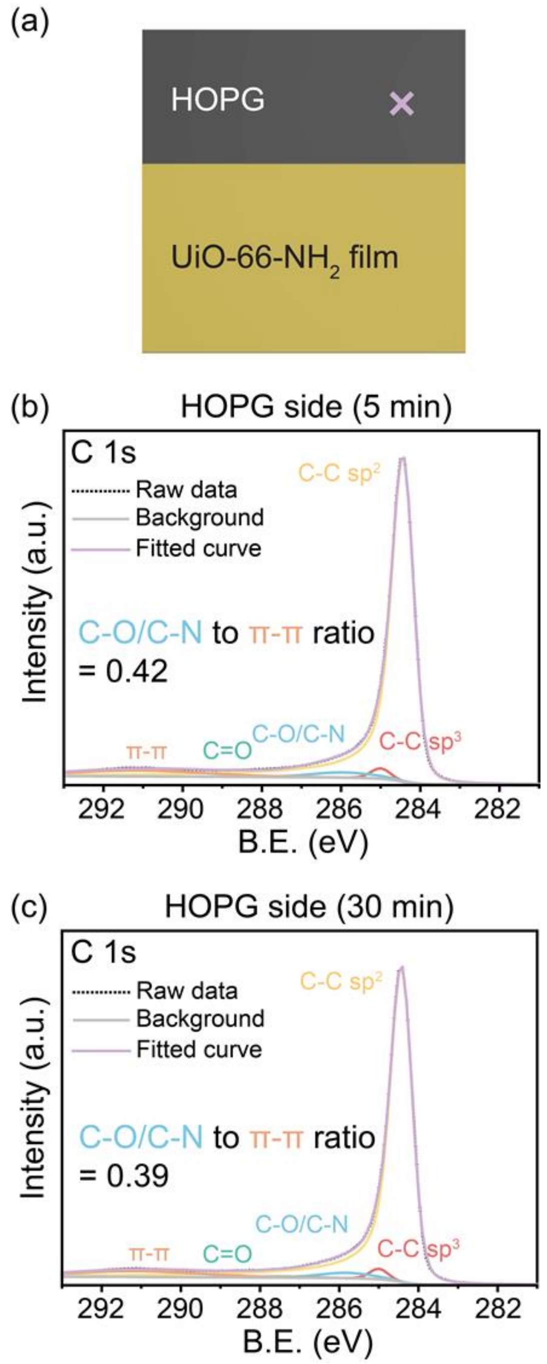

Figure S20 (a) Schematic of the position of the HOPG side. (b) C 1s XPS spectra from HOPG side with a growth time of (b) 5 min and (c) 30 min.

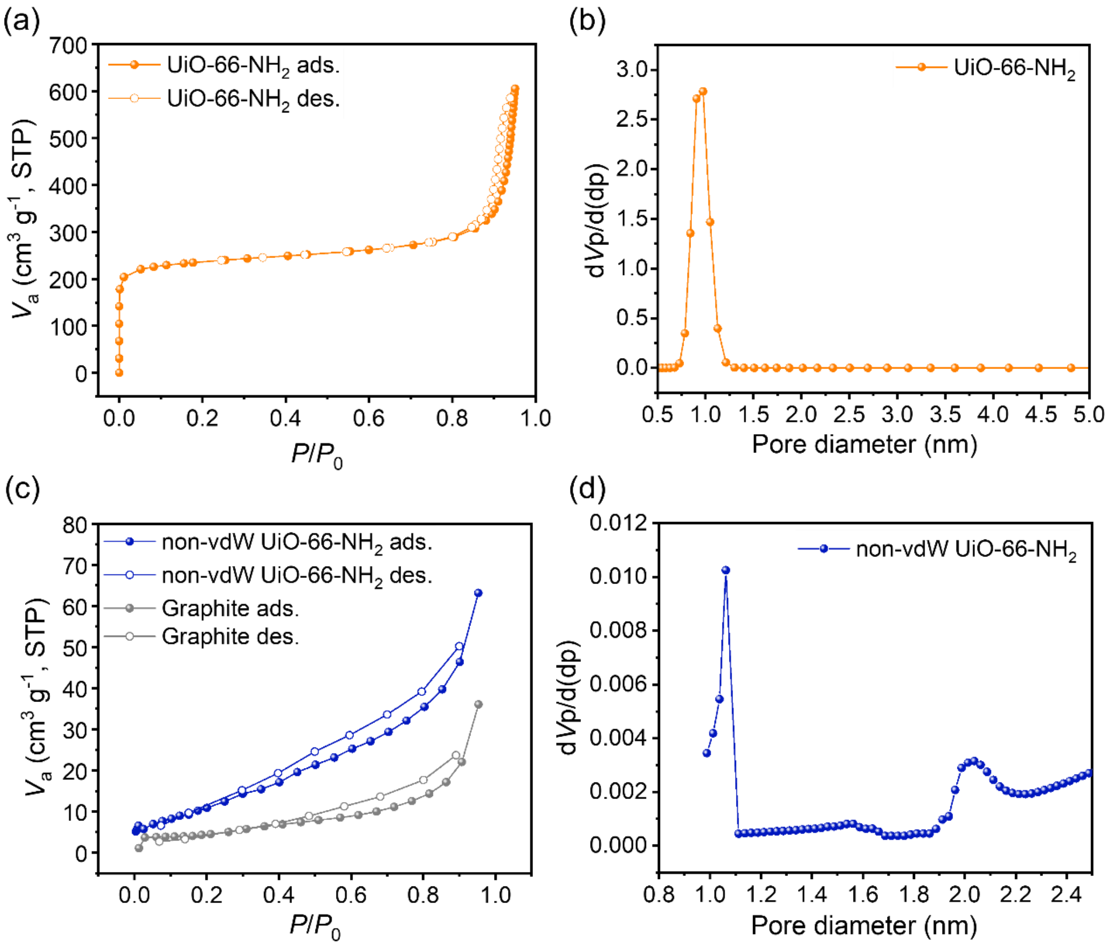

	**UiO-66-NH$_2$**	**Graphite nanoplatelets**	**Non-vdW UiO-66-NH$_2$ @graphite nanoplatelets**	**Non-vdW UiO-66-NH$_2$**
BET surface area (m^2/g)	916.28	16.7	76.51	707.38
Pore volume (cm^3/g)	0.9359	0.056	0.076	0.676
Pore diameter (nm)	1.01	-	1.05	1.05

Figure S21 Nitrogen adsorption data at 77 K for UiO-66-NH$_2$ powder (a), non-vdW 2D UiO-66-NH$_2$ deposited on graphite nanoplatelets, and graphite nanoplatelets (c). The corresponding pore size distributions are shown in (b) for UiO-66-NH$_2$ powder and in (d) for the non-vdW 2D UiO-66-NH$_2$ on graphite nanoplatelets. The table at the bottom summarizes the BET surface area, pore volume, and pore diameter of the samples derived from the adsorption curves.

Supplementary note 5: **GIWAXS measurements**

For the GIWAXS measurement, UiO-66-NH_2 films grown on graphene/SiO_2/Si were prepared by 1 mM Zr^{4+}, 1 mM H_2BDC-NH_2, and a reaction time of 5 min with different cycles. All data were collected at BM01 at the European Synchrotron Radiation Facility (ESRF), with a multipurpose PILATUS2M detector and the wavelength was 1.04157 Å. The size of X-ray beam was ~124 µm × 74 µm. The standard LaB_6 was used to calibrate the X-ray beam before the measurement. During the measurements, the sample was mounted horizontally, and half of the beam was shaded on the sample to ensure the maximum energy can be achieved; therefore the effective beam size was ~124 µm × 37 µm as shown in Figure S23. The combination of rotation of the sample and shift in the sample height was applied to ensure the sample was parallel to the beam, followed by the fine-tuning with a series of ω-scan with 0.01°/step rotating 3° in total to search for the critical angle during the acquisition (Figure S22). The measurement below the critical angle allows the reflection to fully come from the surface (5-10 nm) of samples and the detected intensity is maximized at the critical angle.[42] The optimized measurement incidence angle for non-vdW 2D UiO-66-NH_2 thin films from 1 to 4 cycles was 0.08-0.10°, showing the highest intensity from the sample. The distance from the sample to the detector was 400 mm.

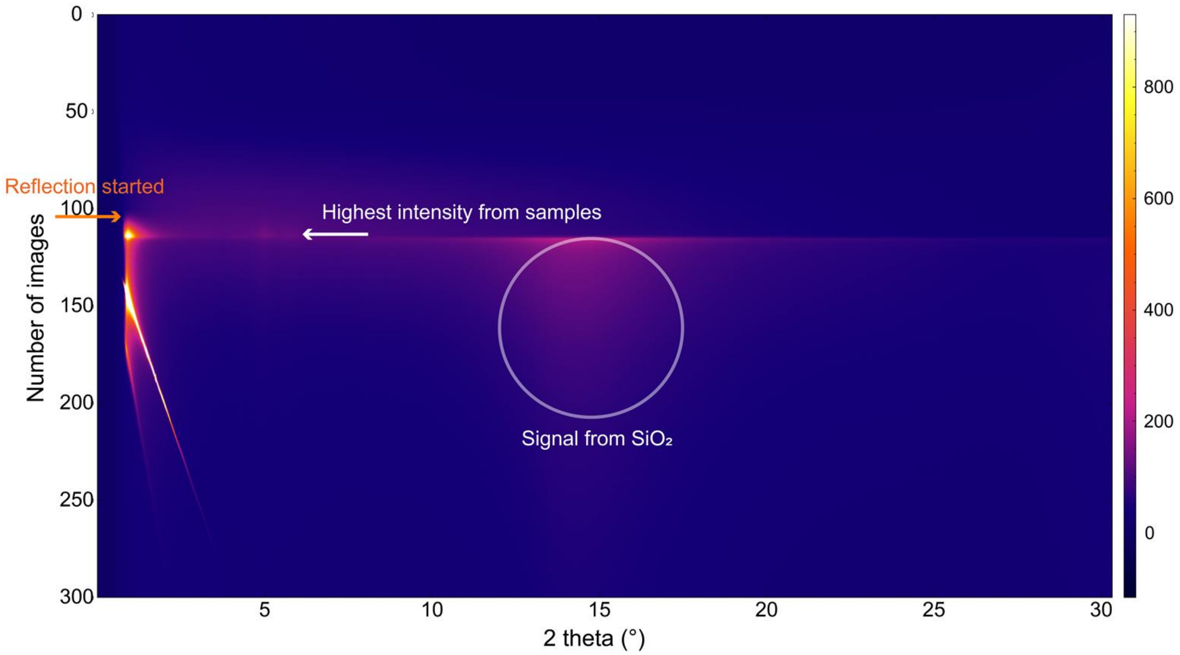

Figure S22 The rotation map shows a series of ω-scans with a 0.01°/step rotation over a total of 3° to determine the critical angle during acquisition. In the process of rotation, the first angle where reflections from the sample were observed was noted at an incidence angle of 0°. The highest intensity observed indicated the critical angle from the samples.

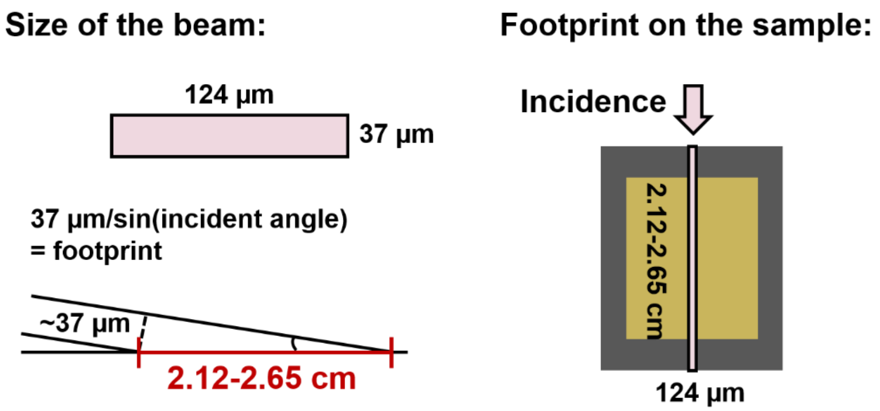

Figure S23 Schematic of the calculation of the footprint on the sample.

Supplementary note 6: Missing wedge in GIWAXS images

The missing wedge in the final GIWAXS images has resulted from the 3D spherical geometry of the Ewald sphere. The Bragg peaks arises when the Ewald sphere intersects with the reciprocal lattice, resulting in the curved surface projecting on the 2D detector. In addition, the final q value was contributed from out-of-plane q_z and in-plane q_x (along beam direction) and q_y since there is always a large contribution from an incident beam, i.e. q_x cannot be zero.[29]

The in-plane contribution was denoted as q_r in the final image and can be described by the following equation (2),[42]

$$q_r = \sqrt{q_x^2 + q_y^2} \neq 0 \tag{2}$$

q_r at zero cannot be assessed in reciprocal space along q_z, resulting in the missing wedge region in the final data with q_z versus q_r.

Supplementary note 7: Analysis of the angle between two lattice planes

Theoretical angle ($\emptyset$) between two lattice planes, (h_1 k_1 l_1) and (h_2 k_2 l_2), were calculated by the following equation for the cubic system, equation (3), which UiO-66-NH_2 belongs to.

$$cos\emptyset = \frac{h_1h_2+k_1k_2+l_1l_2}{\sqrt{h_1^2+k_1^2+l_1^2}\times\sqrt{h_2^2+k_2^2+l_2^2}} \tag{3}$$

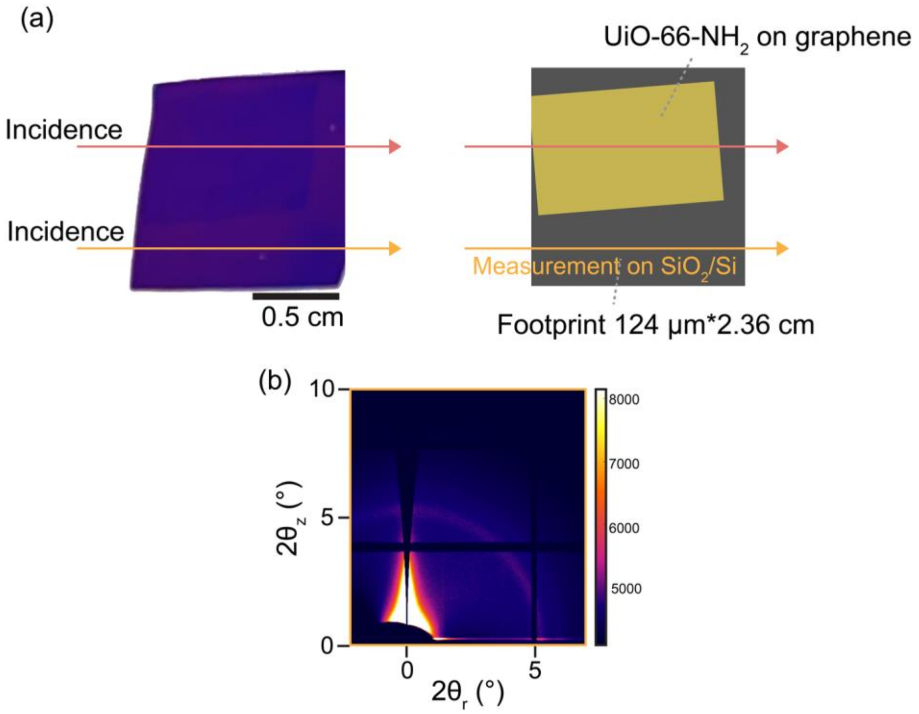

Figure S24 (a) Photograph of the sample of UiO-66-NH_2 fabricated in 5 min for 3 cycles and the schematic shows the sample was shifted to the periphery, devoid of graphene but under identical fabrication conditions as the growth observed on the graphene. (b) GIWAXS measurement from the sample located in the periphery without the graphene at the identical incidence angle.

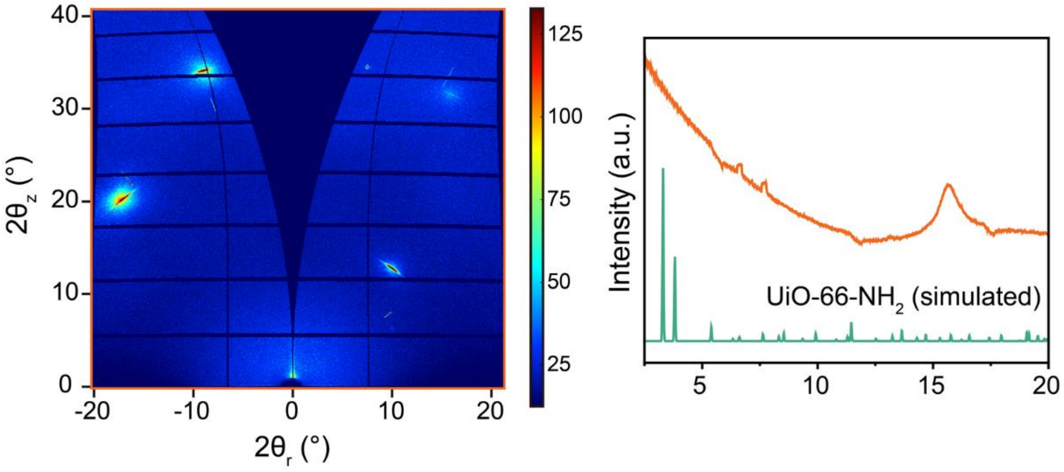

Figure S25 GIWAXS measurement of non-vdW 2D UiO-66-NH_2 synthesized on a single crystal sapphire substrate, grown for 5 minutes over 3 cycles, along with the corresponding 1D patterns obtained at an incidence angle of 0.10° (wavelength = 0.69 Å).

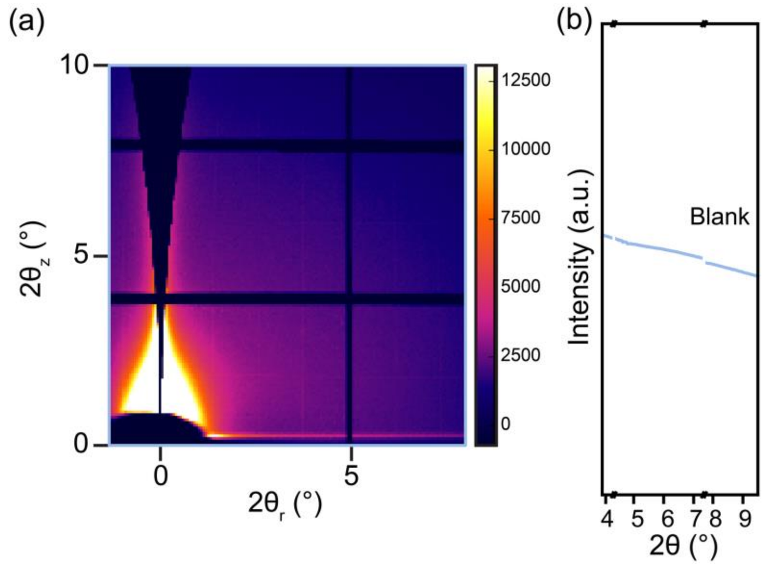

Figure S26 (a) GIWAXS image from graphene/SiO_2/Si and (b) its corresponding 1D patterns at an incidence angle of 0.08°.

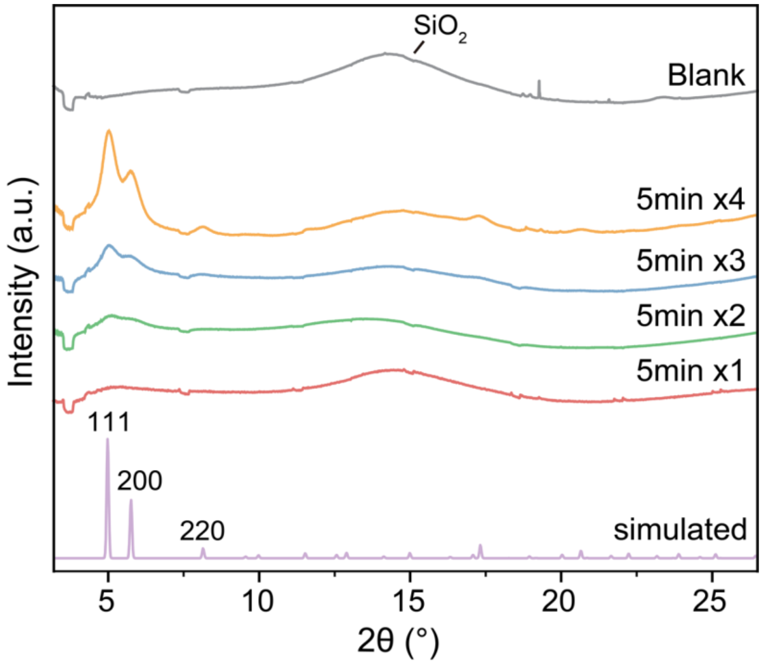

Figure S27 Integrated 1D patterns derived from GIWAXS images representing a wider *q*-range. The notch in the experimental pattern corresponds to the shadow from the detector grid at the synchrotron facility. The impurities were not observed in 1D patterns in wider *q* range.

Supplementary note 8: Lattice mismatch

The lattice mismatch between UiO-66-NH_2 and graphene was calculated using the following equation (4):

$$Lattice\ mismatch = \frac{d_{UiO-66-NH_2} - d_{graphene}}{d_{graphene}} \times 100\% \tag{4}$$

The lattice parameter of UiO-66-NH_2 (a = b = c = 20.75 Å)[40] is much larger than the graphene lattice ($a_1 = a_2$ = 2.46 Å)[43].

Therefore, one lattice of UiO-66-NH_2 and the superlattice of graphene were used to calculate the lattice mismatch. To calculate the lattice mismatch, the smallest lattice of UiO-66-NH_2 ($d_{UiO-66-NH_2}$) was compared with the superlattice of graphene ($d_{graphene}$).

The zone axis along a direction was confirmed by GIWAXS. b and c directions were confirmed through selected area electron diffraction (SAED, Figure S28), which showed that the UiO-66-NH_2 lattice align with the graphene lattice with 0-degree angle between them. This alignment confirms the in-plane orientation of the two materials.

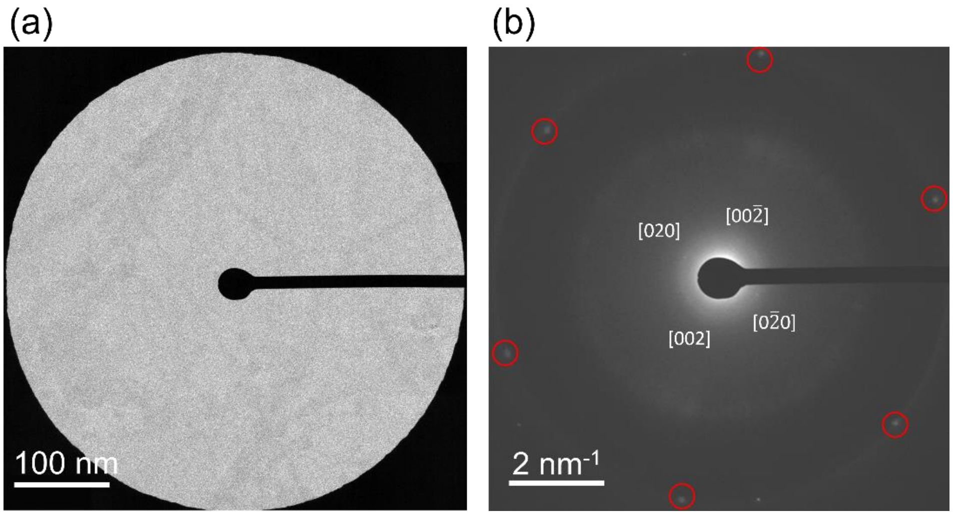

Figure S28 (a) TEM image of the non-vdW 2D UiO-66-NH_2 on graphene and (b) SAED analysis illustrating the orientation of the material. (The diffraction spots from graphene are highlighted by red circles)

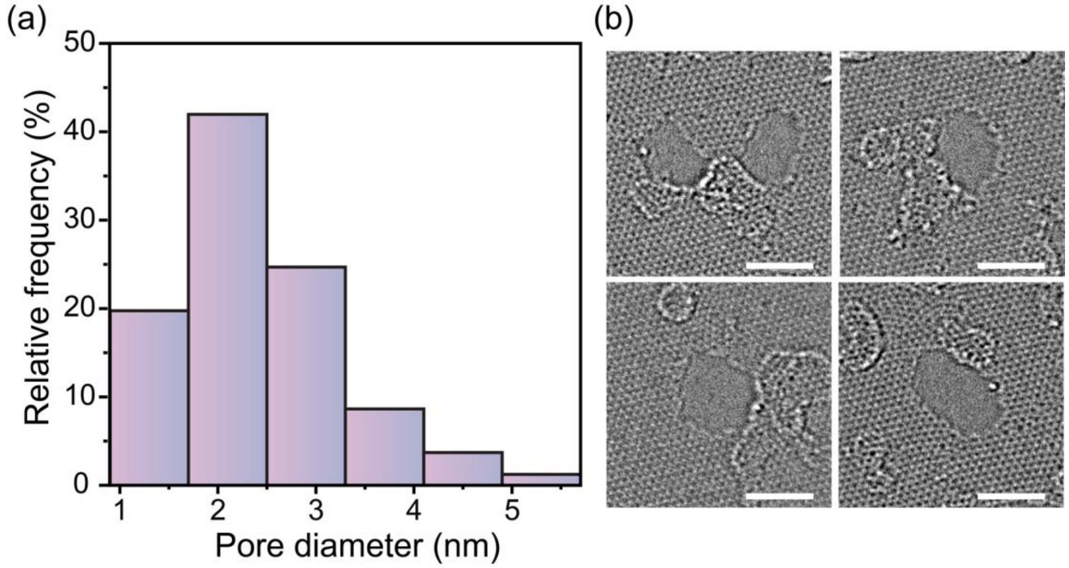

Figure S29 (a) Pore size distribution of porous graphene treated with 5.6 s plasma. (b) AC-HRTEM images of porous graphene showing several pores with sizes around 2 nm. Scale bar: 2 nm.

Supplementary note 9: Ion diffusion measurement

The concentration-driven ion diffusion experiment was conducted using an H-cell, where water naturally diffuses in the opposite direction due to the osmosis pressure. In our system, Nafion significantly reduces water permeance through the membrane, with a measured value of 0.0016 L m^{-2} h^{-1} bar^{-1}.[3] The extremely low water permeance would not affect our measurements, as each ion test requires approximately 6 hours to reach stabilization, making water transport negligible during this period. Nafion is essential in minimizing water crossover from the permeate side to the feed side through forward osmosis, especially the feed side contains a 1 M ion solution. We did not observe significant swelling of Nafion during the measurements, attributed to its thin layer (Figure S30d). Additionally, the UiO-66-NH_2 layer was oriented towards the feed ion solution to prevent dehydration effects from occurring in the supporting layer before reaching the UiO-66-NH_2 film.

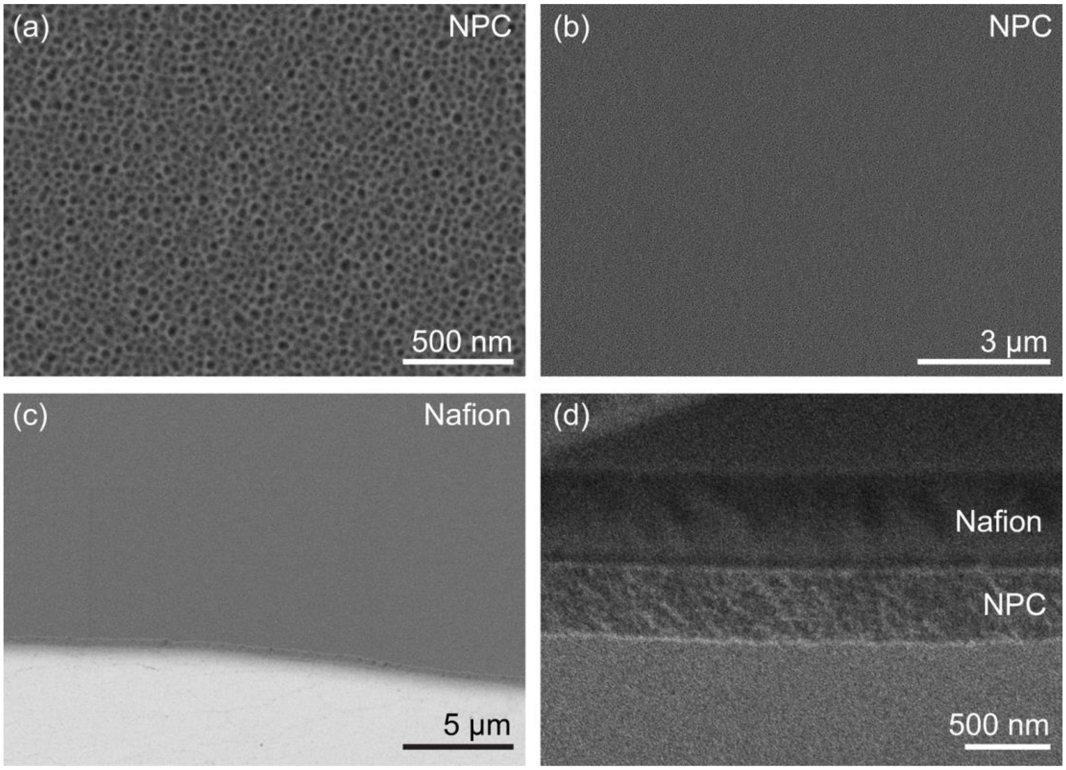

Figure S30 SEM images of NPC layer at (a) high magnification accommodating pore of 20–30 nm and (b) low magnification, and (c) Nafion layer, showing the smoothness of the surface. (d) The cross-section of the supporting layer consists of Nafion and NPC with the thickness of ~500 and 400 nm, respectively.

Supplementary note 10: Synthesis of non-vdW 2D UiO-66 films

The preparation of non-vdW 2D UiO-66 films was similar to that of non-vdW 2D UiO-66-NH_2 films (see experimental section). Briefly, equimolar of 0.5 mM Zr^{4+} and BDC were used to synthesize the non-vdW 2D UiO-66 films, which were successfully formed within 10 min (Figure S31a) and exhibited a thickness of 3.47±0.25 nm (Figure S31b). SEM images confirmed the formation of a continuous and uniform film extending at least over tens of micrometers (Figure S32). The crystallinity of such film was analyzed by GIWAXS, showing the same preferred orientation along 200 plane (Figure S33) for the sample grown 10 min for 3 cycles. Non-vdW 2D UiO-66 film fabricated in 10 min was used to compare the charge effects with UiO-66-NH_2 in ion-ion separation studies.

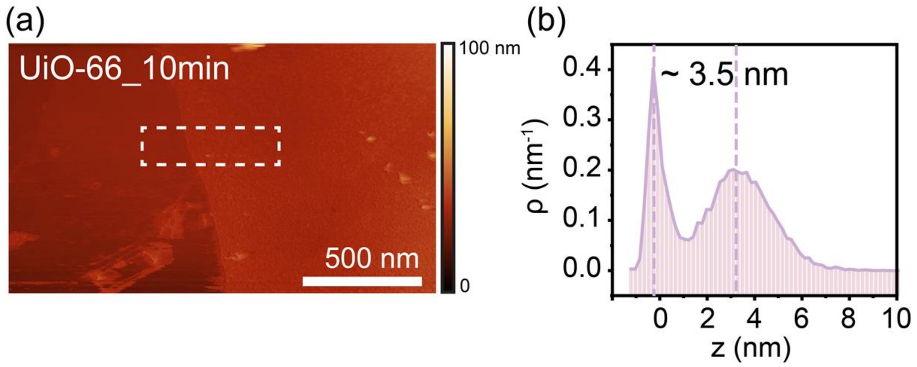

Figure S31 (a) AFM image and (b) the corresponding height profile of 10 min growth of non-vdW 2D UiO-66 film, acquired from the white box in (a).

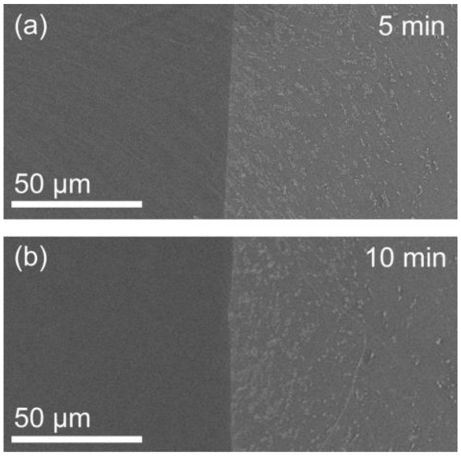

Figure S32 SEM images of non-vdW 2D UiO-66 films: (a) 5 min and (b) 10 min.

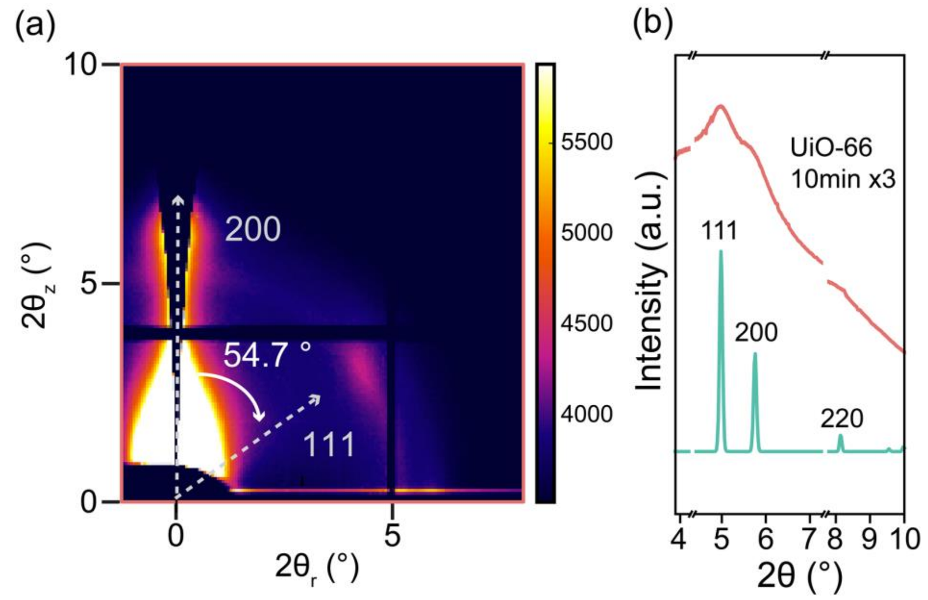

Figure S33 GIWAXS image from non-vdW 2D UiO-66 film prepared in 10 min for three cycles.

Supplementary note 11: Concentration-dependent ion conductance

The formula for estimating the global conductance within a nanopore, represented as G, is given by equation (5):

$$G = \kappa_b \left[\frac{4L}{\pi D^2} \times \frac{1}{1+4\frac{l_{D_u}}{D}} + \frac{2}{\alpha D + \beta l_{D_u}} \right]^{-1} \tag{5}$$

where κ_b, proportional to the salt concentration C_s, denotes the bulk conductivity. In this expression, L refers to the pore length, and D to the pore diameter. The parameters α and β, both set to 2, are approximations derived from a numerical model. The term $l_{D_u} = \kappa_s/\kappa_b \approx \frac{(|\Sigma|/e)}{2C_s}$ represents the Dukhin length, incorporating κ_s as surface conductivity, $|\Sigma|$ as the surface charge, and e as the elementary charge.[44, 45]

In scenarios where l_{D_u} is significantly larger than D, a condition met at low salt concentrations or/and with a high surface charge, the conductance essentially becomes a constant,[31, 32] indicated by equation (6):

$$G(l_{D_u} \to \infty) = \kappa_s \left[\frac{4L}{\pi D} + \frac{2}{\beta} \right]^{-1} \tag{6}$$

This outcome underscores the prediction of conductance saturation under low concentration conditions. Conversely, when the surface charge is absent, $l_{D_u} \to 0$, the conductance is directly proportional to C_s ($\kappa_b \propto C_s$), indicated by equation (7):

$$G\big(l_{D_u} \to 0\big) = \kappa_b \left[\frac{4L}{\pi D^2} + \frac{2}{\alpha D} \right]^{-1} \tag{7}$$

Thus, the conductance dependency on salt concentration is evident. A high surface charge leads to conductance saturation at low concentrations.[44, 45] If there is no contribution from the surface charge, the bulk conductance is proportional to the ion concentration, showing a linear relationship between ion concentration and conductance.

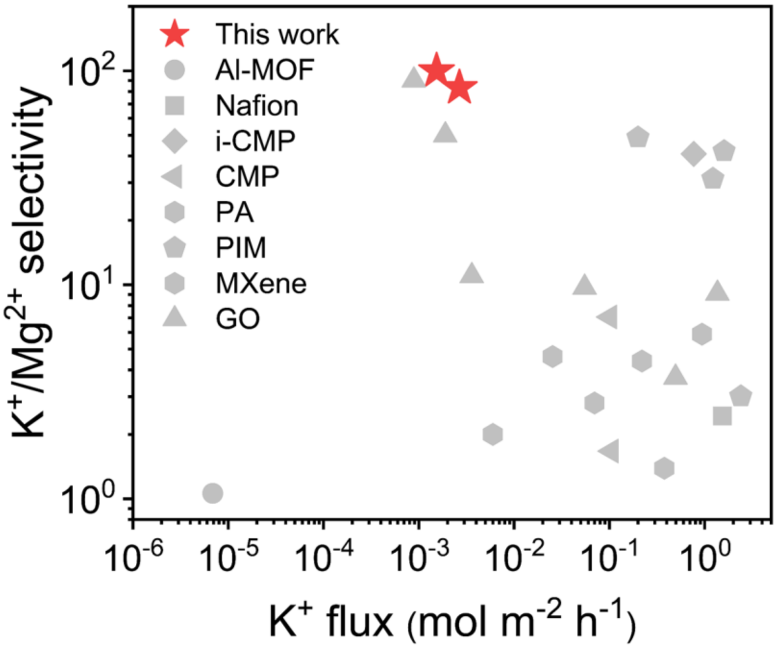

Figure S34 Comparison of the separation performance of the non-vdW 2D UiO-66-NH_2 with other reported membranes (Table S8).

Supplementary note 12: Plasma-treated graphene

The plasma-treated single-layer graphene, etched for 5.6 s, was used as the substrate for membrane fabrication. The plasma-treated graphene resulted in an increased I_D/I_G ratio of 0.761 ± 0.081, indicating successful introduction of defects (Figure S35). The mean pore size of 2.0 nm in the resulting porous graphene was determined based on the AC-HRTEM study (Figure S29). This represents an increase compared to the mean pore size of 1.8 nm observed after 4 s of etching in a previous study.[46] The porous graphene with a much larger pore size than hydrated ion diameter (D_H, 6.6–8.6 Å) is expected to have minimal impact on ion separation selectivity.[3, 47, 48] Thus, the graphene primarily serves as a crystalline substrate, providing crystallographic registry rather than acting as a selective layer for ion separation.

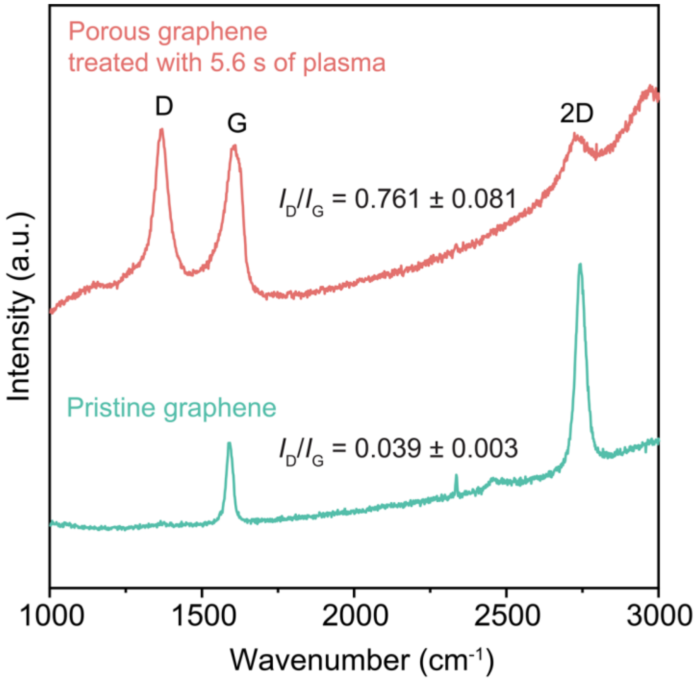

Figure S35 Raman spectra of porous graphene and pristine graphene. Curve fitting for the G and D peaks by Origin was used to determine the I_D/I_G ratio. The average and error bars were derived from three different measurements for both porous and pristine graphene. The pristine graphene synthesized *via* chemical vapor deposition (CVD) exhibited high quality with a low density of intrinsic defects, as evidenced by an I_D/I_G ratio of 0.039 ± 0.003. I_D/I_G ratio, derived from Raman spectra, serves as an indicator of disorder within graphene, with higher values indicating increased defect density.[49]

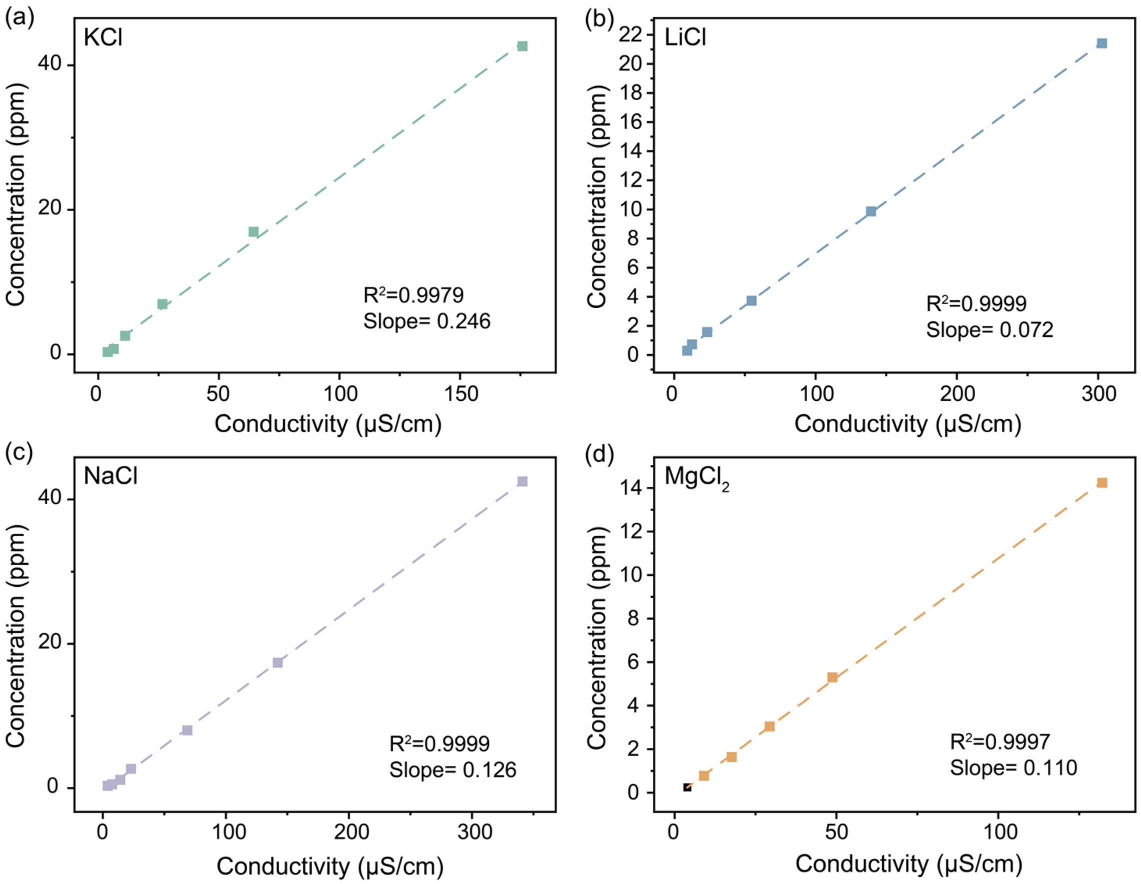

Figure S36 Calibration curves for K^+, Li^+, Na^+, and Mg^{2+} based on ICP.

Reference:

1. Huelsenbeck, L.; Luo, H.; Verma, P.; Dane, J.; Ho, R.; Beyer, E.; Hall, H.; Geise, G. M.; Giri, G., Generalized approach for rapid aqueous MOF synthesis by controlling solution pH. *Cryst. Growth Des.* **2020,** *20* (10), 6787-6795,
2. Huang, S.; Dakhchoune, M.; Luo, W.; Oveisi, E.; He, G.; Rezaei, M.; Zhao, J.; Alexander, D. T.; Züttel, A.; Strano, M. S., Single-layer graphene membranes by crack-free transfer for gas mixture separation. *Nat. Commun.* **2018,** *9* (1), 2632,
3. Zhao, K.; Lee, W.-C.; Rezaei, M.; Chi, H.-Y.; Li, S.; Villalobos, L. F.; Hsu, K.-J.; Zhang, Y.; Wang, F.-C.; Agrawal, K. V., Tuning Pore Size in Graphene in the Angstrom Regime for Highly Selective Ion–Ion Separation. *ACS nano* **2024,** *18* (7), 5571-5580, 10.1021/acsnano.3c11068.
4. Chi, H.-Y. D., A.; Goswami, R.; Song, S., Understanding the crystal structure of metal-organic framework thin film and the structural correlation with the underlying substrates [dataset]. *European Synchrotron Radiation Facility* **2027**, doi.org/10.15151/ESRF-ES-1430229321.
5. Chi, H.-Y. G., R.; Song, S., Understanding the crystal structure of metal-organic framework thin film and the structural correlation with the underlying substrates [dataset]. *European Synchrotron Radiation Facility* **2026**, doi.org/10.15151/ESRF-ES-1315705844.
6. Dong, Y.; Lyu, Q.; Lin, L.-C.; Violet, C.; Lin, B.; Han, Y.; Tang, C.; Yu, H.-Q.; Elimelech, M., Ultrastable ceramic-based metal–organic framework membranes with missing linkers for robust desalination. *Nat. Water* **2024,** *2* (5), 464-474, 10.1038/s44221-024-00218-5.
7. Wang, X.; Lyu, Q.; Tong, T.; Sun, K.; Lin, L.-C.; Tang, C. Y.; Yang, F.; Guiver, M. D.; Quan, X.; Dong, Y., Robust ultrathin nanoporous MOF membrane with intra-crystalline defects for fast water transport. *Nat. Commun.* **2022,** *13* (1), 266,
8. Miyamoto, M.; Hori, K.; Goshima, T.; Takaya, N.; Oumi, Y.; Uemiya, S., An organoselective zirconium- based metal–organic- framework UiO- 66 membrane for pervaporation. *Eur. J. Inorg. Chem.* **2017,** *2017* (14), 2094-2099,
9. Wan, L.; Zhou, C.; Xu, K.; Feng, B.; Huang, A., Synthesis of highly stable UiO-66-NH2 membranes with high ions rejection for seawater desalination. *Microporous Mesoporous Mater.* **2017,** *252*, 207-213, https://doi.org/10.1016/j.micromeso.2017.06.025.
10. Xu, T.; Wu, B.; Li, W.; Li, Y.; Zhu, Y.; Sheng, F.; Li, Q.; Ge, L.; Li, X.; Wang, H., Perfect confinement of crown ethers in MOF membrane for complete dehydration and fast transport of monovalent ions. *Sci. Adv.* **2024,** *10* (19), eadn0944,
11. Li, P.; Sun, Y.; Zhang, Z.; Gu, Z.; Qiao, Z.; Zhong, C., Preparation of UiO-66 membrane through heterogeneous nucleation assisted growth strategy for efficient CO2 capture under humid conditions. *Sep. Purif. Technol.* **2024,** *351*, 128067, https://doi.org/10.1016/j.seppur.2024.128067.
12. Liu, G.; Guo, Y.; Chen, C.; Lu, Y.; Chen, G.; Liu, G.; Han, Y.; Jin, W.; Xu, N., Eliminating lattice defects in metal–organic framework molecular-sieving membranes. *Nat. Mater.* **2023,** *22* (6), 769-776, 10.1038/s41563-023-01541-0.
13. Liu, X.; Demir, N. K.; Wu, Z.; Li, K., Highly water-stable zirconium metal–organic framework UiO-66 membranes supported on alumina hollow fibers for desalination. *J. Am. Chem. Soc.* **2015,** *137* (22), 6999-7002,
14. Xiao, H.; Chai, M.; Hosseini, A.; Korayem, A. H.; Abdollahzadeh, M.; Ahmadi, H.; Chen, V.; Gore, D. B.; Asadnia, M.; Razmjou, A., UiO-66-(COONa) 2 membrane with programmable ionic channels for lithium ion-selective transport. *J. Membr. Sci.* **2023,** *670*, 121312,
15. Guo, H.; Liu, J.; Li, Y.; Caro, J.; Huang, A., Post-synthetic modification of highly stable UiO-66-NH2 membranes on porous ceramic tubes with enhanced H2 separation. *Microporous Mesoporous Mater.* **2021,** *313*, 110823, https://doi.org/10.1016/j.micromeso.2020.110823.
16. Wu, F.; Cao, Y.; Liu, H.; Zhang, X., High-performance UiO-66-NH2 tubular membranes by zirconia-induced synthesis for desulfurization of model gasoline via pervaporation. *J. Membr. Sci.* **2018,** *556*, 54-65, https://doi.org/10.1016/j.memsci.2018.03.090.

17. Friebe, S.; Geppert, B.; Steinbach, F.; Caro, J., Metal–Organic Framework UiO-66 Layer: A Highly Oriented Membrane with Good Selectivity and Hydrogen Permeance. *ACS Appl. Mater. Interfaces* **2017,** *9* (14), 12878-12885, 10.1021/acsami.7b02105.
18. Liu, J.; Canfield, N.; Liu, W., Preparation and Characterization of a Hydrophobic Metal–Organic Framework Membrane Supported on a Thin Porous Metal Sheet. *Ind. Eng. Chem. Res.* **2016,** *55* (13), 3823-3832, 10.1021/acs.iecr.5b04739.
19. Liu, Y.; Liu, L.; Yang, Y.; Yang, T.; Li, C.; Kawi, S.; Wang, X., Salicylaldehyde-assisted ZrO2-induced conversion approach to prepare high performance hollow fiber-supported UiO-66-NH2 membrane for hydrogen separation. *J. Membr. Sci.* **2023,** *684*, 121851, https://doi.org/10.1016/j.memsci.2023.121851.
20. Du, X.-D.; Yi, X.-H.; Wang, P.; Zheng, W.; Deng, J.; Wang, C.-C., Robust photocatalytic reduction of Cr (VI) on UiO-66-NH2 (Zr/Hf) metal-organic framework membrane under sunlight irradiation. *Chem. Eng. J.* **2019,** *356*, 393-399,
21. Zhang, H.; Hou, J.; Hu, Y.; Wang, P.; Ou, R.; Jiang, L.; Liu, J. Z.; Freeman, B. D.; Hill, A. J.; Wang, H., Ultrafast selective transport of alkali metal ions in metal organic frameworks with subnanometer pores. *Sci. Adv.* **2018,** *4* (2), eaaq0066,
22. Shan, B.; James, J. B.; Armstrong, M. R.; Close, E. C.; Letham, P. A.; Nikkhah, K.; Lin, Y.; Mu, B., Influences of deprotonation and modulation on nucleation and growth of UiO-66: Intergrowth and Orientation. *J. Phys. Chem. C* **2018,** *122* (4), 2200-2206,
23. Hod, I.; Bury, W.; Karlin, D. M.; Deria, P.; Kung, C. W.; Katz, M. J.; So, M.; Klahr, B.; Jin, D.; Chung, Y. W., Directed growth of electroactive metal- organic framework thin films using electrophoretic deposition. *Adv. Mater.* **2014,** *26* (36), 6295-6300,
24. Tan, R.; Wang, A.; Malpass-Evans, R.; Williams, R.; Zhao, E. W.; Liu, T.; Ye, C.; Zhou, X.; Darwich, B. P.; Fan, Z., Hydrophilic microporous membranes for selective ion separation and flow-battery energy storage. *Nat. Mater.* **2020,** *19* (2), 195-202,
25. Zhou, Z.; Shinde, D. B.; Guo, D.; Cao, L.; Nuaimi, R. A.; Zhang, Y.; Enakonda, L. R.; Lai, Z., Flexible ionic conjugated microporous polymer membranes for fast and selective ion transport. *Adv. Funct. Mater.* **2022,** *32* (6), 2108672,
26. Zhou, Z.; Guo, D.; Shinde, D. B.; Cao, L.; Li, Z.; Li, X.; Lu, D.; Lai, Z., Precise sub-angstrom ion separation using conjugated microporous polymer membranes. *ACS nano* **2021,** *15* (7), 11970-11980,
27. Gao, S.; Zhu, Y.; Gong, Y.; Wang, Z.; Fang, W.; Jin, J., Ultrathin polyamide nanofiltration membrane fabricated on brush-painted single-walled carbon nanotube network support for ion sieving. *ACS nano* **2019,** *13* (5), 5278-5290,
28. Ren, C. E.; Hatzell, K. B.; Alhabeb, M.; Ling, Z.; Mahmoud, K. A.; Gogotsi, Y., Charge-and size-selective ion sieving through Ti3C2T x MXene membranes. *J. Phys. Chem. Lett.* **2015,** *6* (20), 4026-4031,
29. Wang, J.; Zhang, Z.; Zhu, J.; Tian, M.; Zheng, S.; Wang, F.; Wang, X.; Wang, L., Ion sieving by a two-dimensional Ti3C2T x alginate lamellar membrane with stable interlayer spacing. *Nat. Commun.* **2020,** *11* (1), 3540,
30. Kang, Y.; Hu, T.; Wang, Y.; He, K.; Wang, Z.; Hora, Y.; Zhao, W.; Xu, R.; Chen, Y.; Xie, Z., Nanoconfinement enabled non-covalently decorated MXene membranes for ion-sieving. *Nat. Commun.* **2023,** *14* (1), 4075,
31. Qian, Y.; Shang, J.; Liu, D.; Yang, G.; Wang, X.; Chen, C.; Kou, L.; Lei, W., Enhanced ion sieving of graphene oxide membranes via surface amine functionalization. *J. Am. Chem. Soc.* **2021,** *143* (13), 5080-5090,
32. Zhang, M.; Zhao, P.; Li, P.; Ji, Y.; Liu, G.; Jin, W., Designing biomimic two-dimensional ionic transport channels for efficient ion sieving. *ACS nano* **2021,** *15* (3), 5209-5220,
33. Jian, M.; Qiu, R.; Xia, Y.; Lu, J.; Chen, Y.; Gu, Q.; Liu, R.; Hu, C.; Qu, J.; Wang, H.; Zhang, X., Ultrathin water-stable metal-organic framework membranes for ion separation. *Sci. Adv.* **2020,** *6* (23), eaay3998, doi:10.1126/sciadv.aay3998.

34. Shekhah, O.; Eddaoudi, M., The liquid phase epitaxy method for the construction of oriented ZIF-8 thin films with controlled growth on functionalized surfaces. *Chem. Commun.* **2013,** *49* (86), 10079-10081,
35. Virmani, E.; Rotter, J. M.; Mähringer, A.; Von Zons, T.; Godt, A.; Bein, T.; Wuttke, S.; Medina, D. D., On-surface synthesis of highly oriented thin metal–organic framework films through vapor-assisted conversion. *J. Am. Chem. Soc.* **2018,** *140* (14), 4812-4819,
36. Haraguchi, T.; Otsubo, K.; Sakata, O.; Kawaguchi, S.; Fujiwara, A.; Kitagawa, H., A three-dimensional accordion-like metal–organic framework: synthesis and unconventional oriented growth on a surface. *Chem. Commun.* **2016,** *52* (35), 6017-6020,
37. Falcaro, P.; Okada, K.; Hara, T.; Ikigaki, K.; Tokudome, Y.; Thornton, A. W.; Hill, A. J.; Williams, T.; Doonan, C.; Takahashi, M., Centimetre-scale micropore alignment in oriented polycrystalline metal–organic framework films via heteroepitaxial growth. *Nat. Mater.* **2017,** *16* (3), 342-348,
38. Brundle, C. R.; Crist, B. V., X-ray photoelectron spectroscopy: A perspective on quantitation accuracy for composition analysis of homogeneous materials. *J. Vac. Sci. Technol. A* **2020,** *38* (4), 10.1116/1.5143897.
39. Shearer, G. C.; Chavan, S.; Bordiga, S.; Svelle, S.; Olsbye, U.; Lillerud, K. P., Defect engineering: tuning the porosity and composition of the metal–organic framework UiO-66 via modulated synthesis. *Chem. Mater.* **2016,** *28* (11), 3749-3761,
40. Cavka, J. H.; Jakobsen, S.; Olsbye, U.; Guillou, N.; Lamberti, C.; Bordiga, S.; Lillerud, K. P., A new zirconium inorganic building brick forming metal organic frameworks with exceptional stability. *J. Am. Chem. Soc.* **2008,** *130* (42), 13850-13851,
41. Zhang, D.; Zhu, Y.; Liu, L.; Ying, X.; Hsiung, C.-E.; Sougrat, R.; Li, K.; Han, Y., Atomic-resolution transmission electron microscopy of electron beam–sensitive crystalline materials. *Science* **2018,** *359* (6376), 675-679, doi:10.1126/science.aao0865.
42. Steele, J. A.; Solano, E.; Hardy, D.; Dayton, D.; Ladd, D.; White, K.; Chen, P.; Hou, J.; Huang, H.; Saha, R. A., How to GIWAXS: Grazing Incidence Wide Angle X- Ray Scattering Applied to Metal Halide Perovskite Thin Films. *Adv. Energy Mater.* **2023,** *13* (27), 2300760,
43. Hembacher, S.; Giessibl, F. J.; Mannhart, J.; Quate, C. F., Revealing the hidden atom in graphite by low-temperature atomic force microscopy. *Proc. Natl. Acad. Sci.* **2003,** *100* (22), 12539-12542,
44. Lee, C.; Joly, L.; Siria, A.; Biance, A.-L.; Fulcrand, R.; Bocquet, L., Large Apparent Electric Size of Solid-State Nanopores Due to Spatially Extended Surface Conduction. *Nano Lett.* **2012,** *12* (8), 4037-4044, 10.1021/nl301412b.
45. Feng, J.; Graf, M.; Liu, K.; Ovchinnikov, D.; Dumcenco, D.; Heiranian, M.; Nandigana, V.; Aluru, N. R.; Kis, A.; Radenovic, A., Single-layer MoS2 nanopores as nanopower generators. *Nature* **2016,** *536* (7615), 197-200,
46. He, G.; Huang, S.; Villalobos, L. F.; Zhao, J.; Mensi, M.; Oveisi, E.; Rezaei, M.; Agrawal, K. V., High-permeance polymer-functionalized single-layer graphene membranes that surpass the postcombustion carbon capture target. *Energy Environ. Sci.* **2019,** *12* (11), 3305-3312,
47. Esfandiar, A.; Radha, B.; Wang, F.; Yang, Q.; Hu, S.; Garaj, S.; Nair, R. R.; Geim, A.; Gopinadhan, K., Size effect in ion transport through angstrom-scale slits. *Science* **2017,** *358* (6362), 511-513,
48. Gopinadhan, K.; Hu, S.; Esfandiar, A.; Lozada-Hidalgo, M.; Wang, F.; Yang, Q.; Tyurnina, A.; Keerthi, A.; Radha, B.; Geim, A., Complete steric exclusion of ions and proton transport through confined monolayer water. *Science* **2019,** *363* (6423), 145-148,
49. Ferrari, A. C.; Basko, D. M., Raman spectroscopy as a versatile tool for studying the properties of graphene. *Nat. Nanotechnol.* **2013,** *8* (4), 235-246,
